# Integrative multi-omics analysis reveals novel idiopathic pulmonary fibrosis endotypes associated with disease progression

**DOI:** 10.1186/s12931-023-02435-0

**Published:** 2023-05-31

**Authors:** Peifeng Ruan, Jamie L Todd, Hongyu Zhao, Yi Liu, Richard Vinisko, Julia F. Soellner, Ramona Schmid, Robert J. Kaner, Tracy R. Luckhardt, Megan L. Neely, Imre Noth, Mary Porteous, Rishi Raj, Zeenat Safdar, Mary E Strek, Christian Hesslinger, Scott M. Palmer, Thomas B. Leonard, Margaret L. Salisbury

**Affiliations:** 1 Department of Biostatistics, Yale School of Public Health, New Haven, CT USA; 2 Duke Clinical Research Institute, Durham, NC USA; 3 Duke University Medical Center, Durham, NC USA; 4 Boehringer Ingelheim Pharmaceuticals, Inc, Ridgefield, CT USA; 5 Boehringer Ingelheim Pharma GmbH & Co. KG, Biberach, Germany; 6 Weill Cornell Medicine, New York, NY USA; 7 Department of Medicine, University of Alabama at Birmingham, Birmingham, AL USA; 8 Division of Pulmonary and Critical Care Medicine, University of Virginia, Charlottesville, VA USA; 9 Hospital of the University of Pennsylvania, Philadelphia, PA USA; 10 Stanford University School of Medicine, Stanford, CA USA; 11 Houston Methodist Lung Center, Houston, TX USA; 12 Section of Pulmonary and Critical Care Medicine, University of Chicago, Chicago, IL USA; 13 Department of Medicine, Vanderbilt University Medical Center, 1211 Medical Center Drive, 37232 Nashville, TN USA

**Keywords:** Lung fibrosis, Cluster analysis, Biomarkers, Proteomics, RNA, Computational biology

## Abstract

**Background:**

Idiopathic pulmonary fibrosis (IPF) is characterized by the accumulation of extracellular matrix in the pulmonary interstitium and progressive functional decline. We hypothesized that integration of multi-omics data would identify clinically meaningful molecular endotypes of IPF.

**Methods:**

The IPF-PRO Registry is a prospective registry of patients with IPF. Proteomic and transcriptomic (including total RNA [toRNA] and microRNA [miRNA]) analyses were performed using blood collected at enrollment. Molecular data were integrated using Similarity Network Fusion, followed by unsupervised spectral clustering to identify molecular subtypes. Cox proportional hazards models tested the relationship between these subtypes and progression-free and transplant-free survival. The molecular subtypes were compared to risk groups based on a previously described 52-gene (toRNA expression) signature. Biological characteristics of the molecular subtypes were evaluated via linear regression differential expression and canonical pathways (Ingenuity Pathway Analysis [IPA]) over-representation analyses.

**Results:**

Among 232 subjects, two molecular subtypes were identified. Subtype 1 (n = 105, 45.3%) and Subtype 2 (n = 127, 54.7%) had similar distributions of age (70.1 +/- 8.1 vs. 69.3 +/- 7.6 years; p = 0.31) and sex (79.1% vs. 70.1% males, p = 0.16). Subtype 1 had more severe disease based on composite physiologic index (CPI) (55.8 vs. 51.2; p = 0.002). After adjusting for CPI and antifibrotic treatment at enrollment, subtype 1 experienced shorter progression-free survival (HR 1.79, 95% CI 1.28,2.56; p = 0.0008) and similar transplant-free survival (HR 1.30, 95% CI 0.87,1.96; p = 0.20) as subtype 2. There was little agreement in the distribution of subjects to the molecular subtypes and the risk groups based on 52-gene signature (kappa = 0.04, 95% CI= -0.08, 0.17), and the 52-gene signature risk groups were associated with differences in transplant-free but not progression-free survival. Based on heatmaps and differential expression analyses, proteins and miRNAs (but not toRNA) contributed to classification of subjects to the molecular subtypes. The IPA showed enrichment in pulmonary fibrosis-relevant pathways, including mTOR, VEGF, PDGF, and B-cell receptor signaling.

**Conclusions:**

Integration of transcriptomic and proteomic data from blood enabled identification of clinically meaningful molecular endotypes of IPF. If validated, these endotypes could facilitate identification of individuals likely to experience disease progression and enrichment of clinical trials.

**Trial registration:**

NCT01915511

**Supplementary Information:**

The online version contains supplementary material available at 10.1186/s12931-023-02435-0.

## Background

Idiopathic pulmonary fibrosis (IPF) is characterized by abnormal accumulation of extracellular matrix (ECM) in the pulmonary interstitium. The natural history of IPF is characterized by progressive decline in lung function, often culminating in death from respiratory failure [1]. Significant progress has been made in understanding the pathobiology of IPF, including high-throughput assessments of genetic risk factors, changes in gene expression, and alterations in the abundance of proteins in the lungs or peripheral blood that are associated with the development or progression of IPF [2–11]. These studies reinforce the conceptualization of IPF as a disease initiated by recurrent, low-grade injury to the lung epithelium, with pathologic disruption in cellular aging and innate immune responses to injury as drivers of ECM deposition. Molecular analyses have yielded candidate biomarkers for confirmation of an IPF diagnosis or staging of disease. A 52-gene expression signature [9, 10] and, more recently, a 13-gene signature developed using unsupervised clustering [12], identified persons with IPF at high risk of mortality when measured in peripheral blood. In addition, a signature of 17 proteins measured in peripheral blood identified patients with non-idiopathic pulmonary fibrosis who were at high risk of disease progression [11]. MicroRNAs (miRNAs), small non-coding RNAs that regulate gene expression post-transcription [13], have been mechanistically linked to IPF, but are incompletely studied as biomarkers of disease progression [14–16].

Integrative multi-omics analyses have identified molecular subtypes of several forms of cancer [17–21]. With a few exceptions, high-throughput studies of the molecular landscape of IPF have focused on alterations in a single type of molecule [22–25]. In this study, we measured the abundance of proteins and the expression of total RNA and miRNAs in the peripheral blood of patients with IPF enrolled in a multi-center observational registry. We hypothesized that cross-platform integration and simultaneous assessment of several types of molecules in the gene-to-function pathway using an unsupervised integrative clustering method would identify clinically meaningful molecular endotypes of IPF.

## Methods

This study included 300 patients enrolled in the US-based, multi-center, observational Idiopathic Pulmonary Fibrosis Prospective Outcomes (IPF-PRO) Registry (NCT01915511), whose key inclusion criterion is IPF diagnosed or confirmed at the enrolling center in the past 6 months [26]. Enrollment procedures included collection of a blood sample, demographics and health information. Patients are followed longitudinally with information such as pulmonary function tests collected as part of their routine clinical care. For this analysis, all participants who were enrolled between June 2014 and February 2017, with longitudinal outcomes ascertained through December 2019, and who had blood samples available for molecular analyses described below were selected. A formal power analysis was not conducted.

### Identification of molecular subtypes of IPF

Whole blood and plasma collected at enrollment were stored centrally. The process used to quantify plasma proteins by aptamer-based methods, measure miRNA expression in plasma, sequence total RNA (referred to as toRNA) in whole blood, and to perform bioinformatics analyses, is described in Additional file 1: Section S1. After excluding 52 subjects due to low toRNA quality, 9 due to low miRNA quality, and 7 for low quality of both, data from 232 subjects were analyzed. Additional file 2: Table S1 summarizes the features within each molecule type (toRNA, miRNA, protein) that were available for modeling.

To identify molecular subtypes of IPF, an integrative, two-step method, spectral clustering Similarity Network Fusion (scSNF) was used to cluster subjects based on data from all three molecule types. First, Similarity Network Fusion, a method that integrates similarity networks, was applied to fuse proteomics, miRNA and toRNA expression data for each subject [27]. Second, an unsupervised spectral clustering method [28] was applied to the fused similarity network. This method uses eigenvectors of the graph Laplacian of the similarity network to cluster subjects. To achieve stable clustering results, consensus clustering with 100 iterations and a 0.8 subsampling ratio was applied [29]. Average Silhouette scores [30] were used to determine the number of clusters, with 2 to 10 clusters assessed, where scores near 1 indicate optimal clustering and a decrease toward 0 indicates increasing overlap in clusters. The scSNF method was compared to the alternative integrative clustering methods iCluster+ [31] and iClusterBayes [32]. Sensitivity analyses assessed clustering membership when the toRNA variance filter (set as the top 10% most variable features for the main analysis) was adjusted to include the top 50% most variable features or to 100%.

### Clinical characterization of molecular subtypes of IPF

Patient characteristics within each molecular subtype were summarized using means and standard deviations for continuous variables and numbers and percentages for categorical variables. Characteristics were compared between the subtypes using the Kruskal-Wallis test for continuous variables and the Chi-square test for categorical variables, with p < 0.05 considered statistically significant. To determine the relationship between the molecular subtypes and clinically meaningful outcomes, the occurrence of two composite outcomes was determined: (1) lung transplant or death; and (2) disease progression, defined as ≥ 10% absolute decline in forced vital capacity (FVC) % predicted, lung transplant, or death. FVC decline recorded at the first instance of a post-enrollment value ≥ 10% lower than the enrollment value. These events were selected because they can be objectively assessed and have similar importance in the natural history of IPF [33]. Transplant-free and progression-free survival were considered separately to enable an evaluation of the relationship between molecular markers and these two outcomes. Subjects who withdrew from the study or had not experienced an outcome by December 2019 were censored on the date of their last follow-up visit. Kaplan-Meier plots and Cox proportional hazards regression models (unadjusted, and adjusted for baseline disease severity based on the composite physiologic index [CPI] [34] and antifibrotic treatment status at enrollment) were used to determine the risk of the two composite outcomes.

Next, to place the molecular subtypes identified in this study in the context of existing literature, the previously described 52-gene expression signature was applied to group the subjects as high-risk or low-risk (for death or transplant) [9, 10]. Cohen’s kappa assessed agreement in groupings based on the molecular subtypes and the 52-gene signature. The risk of experiencing each composite outcome was assessed for the 52-gene signature high-risk compared to the low-risk group using Kaplan-Meier plots and Cox proportional hazards regression models (unadjusted, and adjusted for CPI and antifibrotic treatment status).

### Biological characterization of molecular subtypes of IPF

The molecular characteristics that distinguished the subtypes identified by scSNF were investigated in several ways. First, Normalized Mutual Information (NMI) measured agreement of distribution of the subjects to the subtypes when clusters were formed using only one data type (i.e., protein, toRNA, or miRNA) compared to using all three data types [35]. Next, heatmaps visualized differences in protein abundance, toRNA, and miRNA expression between the subtypes. Finally, a random forest model [36] with 5-fold cross validation was used to identify molecular features that could classify individuals to a subtype. The classifier process is described in Additional file 3: Section S2 and Figure S1.

To investigate the biology underlying the molecular subtypes, linear regression models identified differentially expressed features within each molecule set (see Additional file 4: Section S3 for details). Then, Ingenuity Pathway Analysis (IPA) (QIAGEN Inc.) [37] identified canonical pathways in which differentially expressed features were significantly over-represented, based on a hypergeometric/right-tailed Fisher’s exact test with false discovery rate (FDR)-adjusted p-value < 0.05 [38]. IPA analyses were conducted separately among significantly up-regulated and down-regulated features. All analyses except those performed with IPA were performed using R version 3.6.1.

## Results

### Unsupervised clustering identified molecular subtypes of IPF with distinct clinical characteristics

The scSNF clustering method suggested two as the optimal number of clusters based on Silhouette scores (Additional file 5: Figure S2). The alternative clustering methods (iCluster + and iClusterBayes) suggested a larger number of clusters, but these were largely overlapping with the scSNF clusters (Additional file 5: Section S4 and Table S2). In the sensitivity analyses based on different RNA-seq variance filters, scSNF clusters were preserved across variance filtering cutpoints (Additional file 5: Table S3).

Subtype 1 comprised 105 (45.3%) subjects while subtype 2 comprised 127 (54.7%) subjects. Subtype 1 had more severe disease at baseline, with lower diffusion capacity of the lung for carbon monoxide (DLco) % predicted (38.3 vs. 43.1; p = 0.01), lower FVC % predicted (67.9 vs. 73.8; p = 0.02), and higher CPI (55.8 vs. 51.2; p = 0.002). There were no significant differences in age, sex, smoking status, medical history, GAP stage [39], diagnostic category [40], or antifibrotic treatment status at enrollment (Table 1).

**Table 1:** Patient characteristics at enrollment by molecular subtype

	Subtype 1 (N = 105)	Subtype 2 (N = 127)	p-value^+^
Age	70.08 (8.07)	69.33 (7.62)	0.31
Sex			0.16
M	83 (79.1%)	89 (70.1%)	
F	22 (21.0%)	38 (30.0%)	
Ever smoker			0.59
Y	73 (69.5%)	83 (65.4%)	
N	32 (30.5%)	44 (34.7%)	
History of coronary artery disease (including prior MI)*			1.0
Y	33 (31.4%)	39 (31.0%)	
N	72 (68.6%)	87 (69.1%)	
History of COPD*			1.0
Y	19 (18.1%)	22 (17.5%)	
N	86 (81.9%)	104 (82.5%)	
History of diabetes*			0.18
Y	24 (22.9%)	19 (15.1%)	
N	81 (77.1%)	107 (84.9%)	
Antifibrotic treatment			0.07
Nintedanib	24 (22.9%)	18 (14.17%)	
Pirfenidone	41 (38.1%)	43 (33.86%)	
Neither	40 (39.1%)	66 (52.0%)	
DL_CO_ % predicted	38.31 (12.46)	43.09 (14.69)	0.01
FVC % predicted	67.89 (15.18)	73.82 (17.72)	0.02
FEV_1_% predicted	76.09 (16.11)	81.03 (20.00)	0.12
CPI [34]	55.77 (9.93)	51.21 (11.49)	0.01
Diagnostic category [40]			0.41
Definite IPF	77 (73.3%)	95 (74.8%)	
Probable IPF	22 (21.0%)	29 (22.8%)	
Possible IPF	6 (5.7%)	3 (2.4%)	
GAP stage [39]			0.15
1	22 (21.0%)	40 (31.5%)	
2	64 (61.0%)	71 (55.9%)	
3	19 (18.1%)	16 (12.6%)	

Data are mean (SD) or n (%). Abbreviations: MI: Myocardial infarction, DLco: Diffusing capacity of the lungs for carbon monoxide, FVC: Forced vital capacity, FEV_1_: Forced expiratory volume in the first second, CPI: Composite physiologic index.

*One subject in subtype 1 was missing medical history.

^+^Kruskal-Wallis test compared continuous variables and Chi-square test compared categorical variables.

**Fig. 1:**
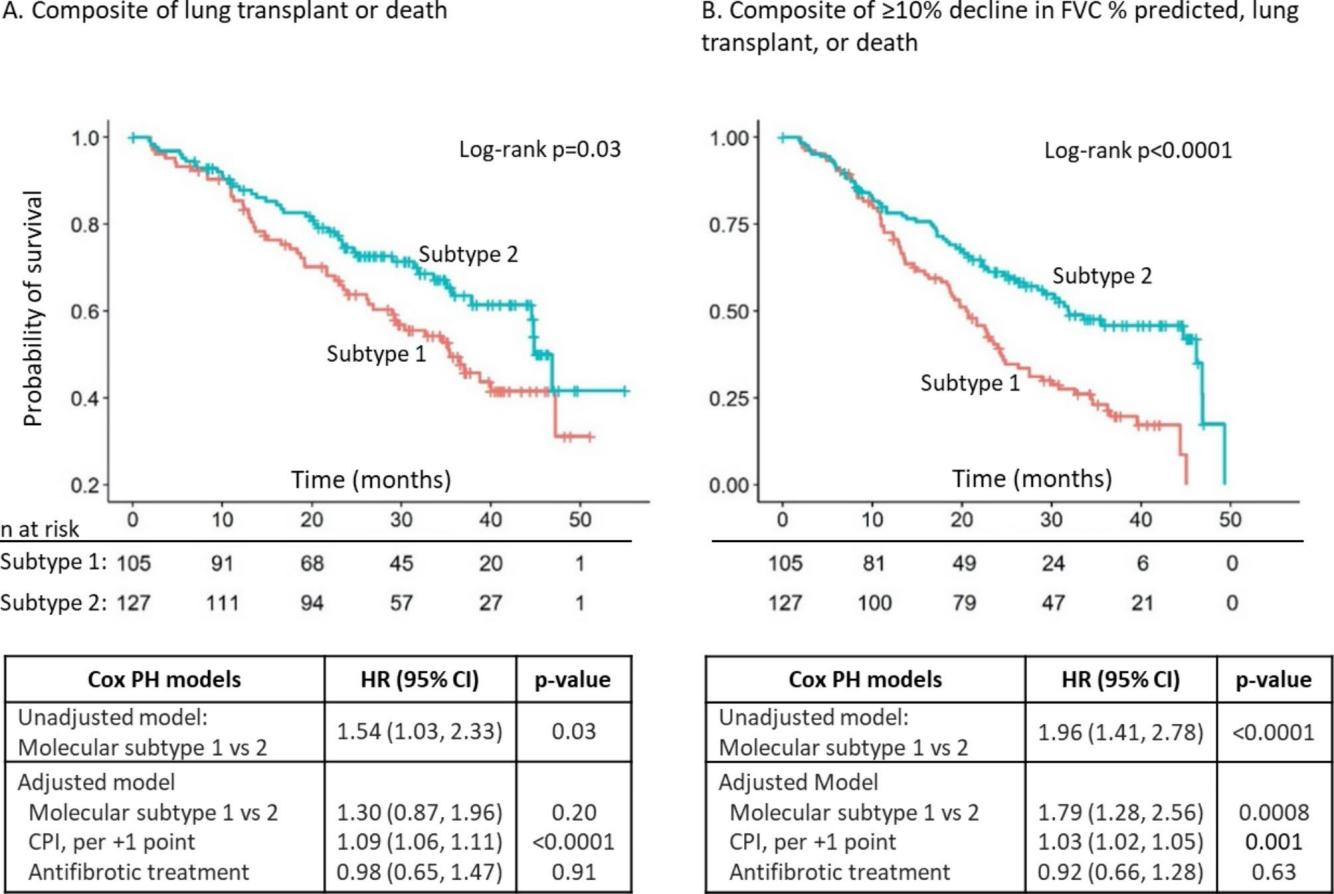
Risk of outcomes based on the molecular IPF subtype. Kaplan-Meier plots show the time from enrollment to the composite outcome of lung transplant or death (**A**) and the composite outcome of ≥ 10% absolute decline in FVC % predicted, lung transplant, or death (**B**) for subtype 1 compared to subgroup 2. The associated tables show the unadjusted hazard ratio and the hazard ratio adjusted for CPI and antifibrotic treatment use for subtype 1 compared to subtype 2. HR: hazard ratio; PH: proportional hazards; CPI: composite physiologic index

During a median follow-up of 27.5 months (interquartile range 15.8–36.7 months), the composite of lung transplant or death occurred in 95 (40.9%) subjects (18 lung transplants, 77 deaths, and 137 censored non-events). The composite of disease progression occurred in 143 (61.6%) subjects (88 with FVC decline, 8 lung transplants, 47 deaths, and 89 censored non-events). In the unadjusted analysis, subjects in subtype 1 experienced a significantly shorter time to lung transplant or death (median 35 vs. 45 months, log-rank p = 0.03; HR 1.54, 95% CI 1.03–2.23, p = 0.03) (Fig. 1A) and a significantly shorter time to disease progression (median 21 vs. 32 months, log-rank p < 0.0001; HR 1.96, 95% CI 1.41, 2.78, p < 0.0001) (Fig. 1B). After adjusting for CPI and antifibrotic treatment status at enrollment, subtype 1 had a significantly shorter time to disease progression (adjusted HR 1.79, 95% CI 1.28, 2.56; p = 0.0008) but no different time to transplant or death (adjusted HR 1.30, 95% CI 0.87, 1.96; p = 0.20) (Fig. 1).

**Fig. 2:**
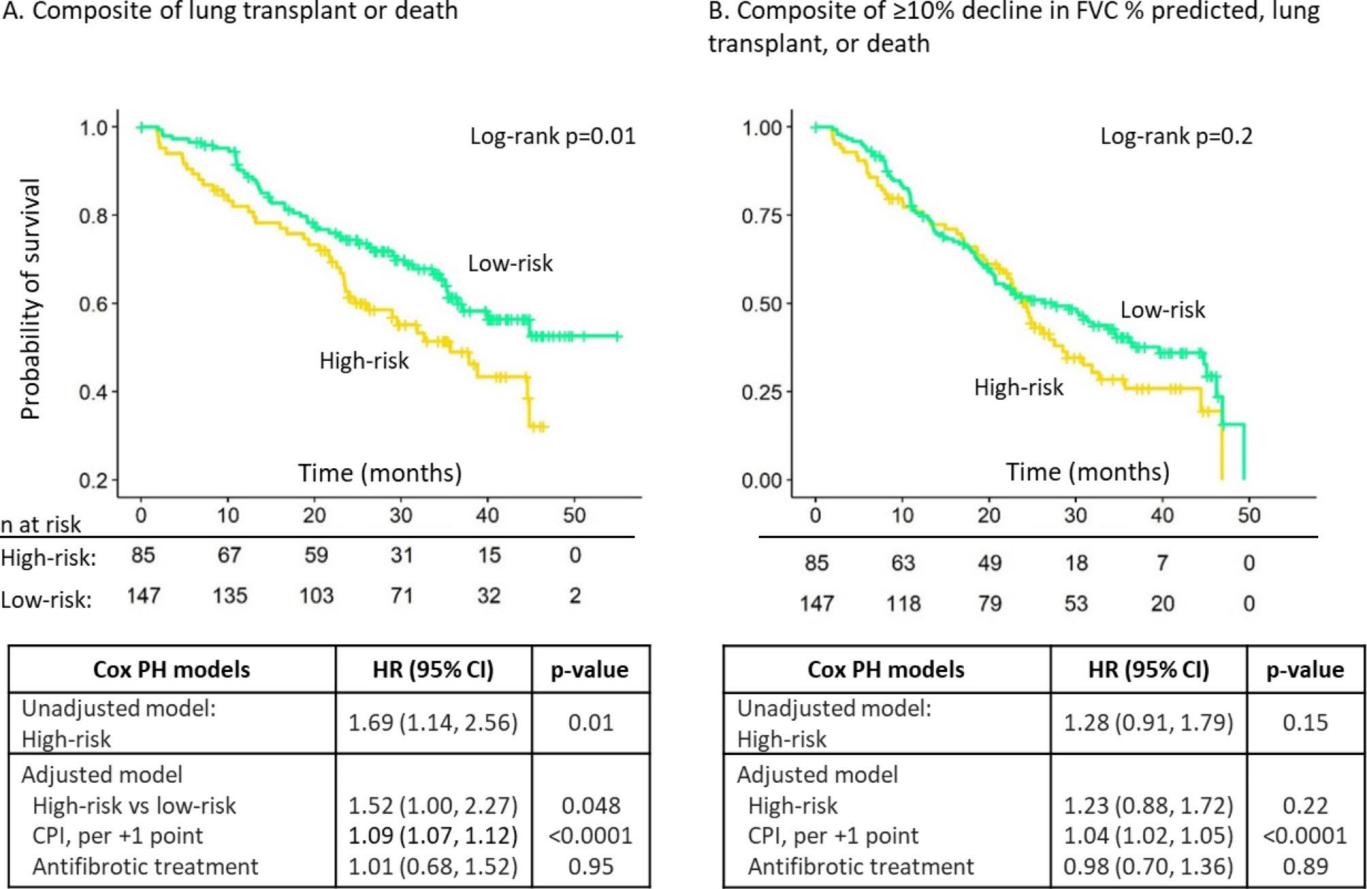
Risk of outcomes based on the 52-gene signature. Kaplan-Meier plots show the time from enrollment to the composite outcome of lung transplant or death (**A**) and the composite outcome of ≥ 10% absolute decline in FVC % predicted, lung transplant, or death (**B**) for the high-risk group and low-risk group. The associated tables show the unadjusted hazard ratio and the hazard ratio adjusted for CPI and antifibrotic treatment use for the high-risk group compared to the low-risk group. HR: hazard ratio; PH: proportional hazards; CPI: composite physiologic index

When the 52-gene signature was applied to our analysis cohort, the high-risk group comprised 85 (36.6%) subjects and the low-risk group 147 (63.3%) subjects. The molecular subtypes were distinct from the 52-gene signature risk groups, with no agreement beyond chance (k = 0.04, 95% CI= -0.08, 0.17), p = 0.49; Additional file 5: Table S4). The 52-gene high-risk group experienced an increased risk of lung transplant or death in unadjusted and adjusted models (Fig. 2A). However, the high-risk group did not experience a significantly increased risk for disease progression in unadjusted or adjusted models (Fig. 2B).

### Molecular subtypes differed based on proteomics and miRNA features

**Fig. 3:**
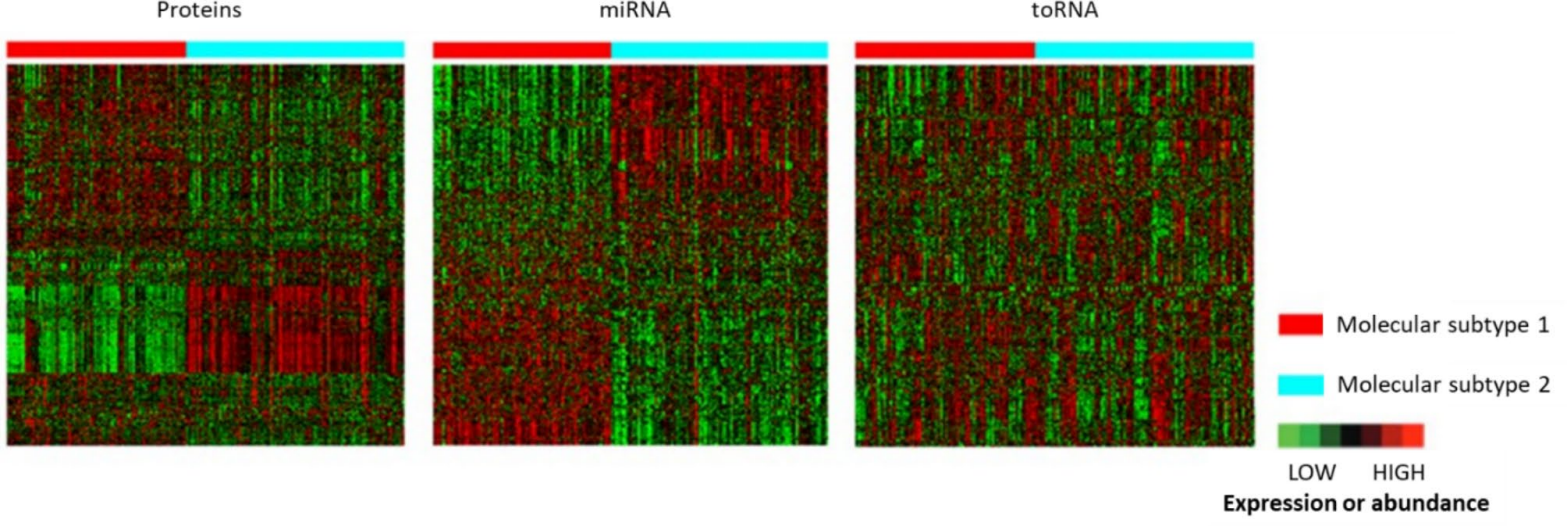
Heatmaps comparing protein abundance and miRNA or toRNA expression in the molecular IPF subtypes determined by the spectral clustering Similarity Network Fusion (scSNF) integrated two-step method

Based on good agreement (indicating substantial overlap) on the distribution of subjects to a cluster using a single molecule type compared to the scSNF multi-omics data, proteins (NMI = 0.41) and miRNAs (NMI = 0.62) contributed substantially to the clustering, while toRNAs had little effect (NMI = 0.0003) (Additional file 5: Table S5). Heatmaps confirmed this assessment, with clear differences between the subtypes for proteins and miRNAs but not for toRNAs (Fig. 3).

**Fig. 4:**
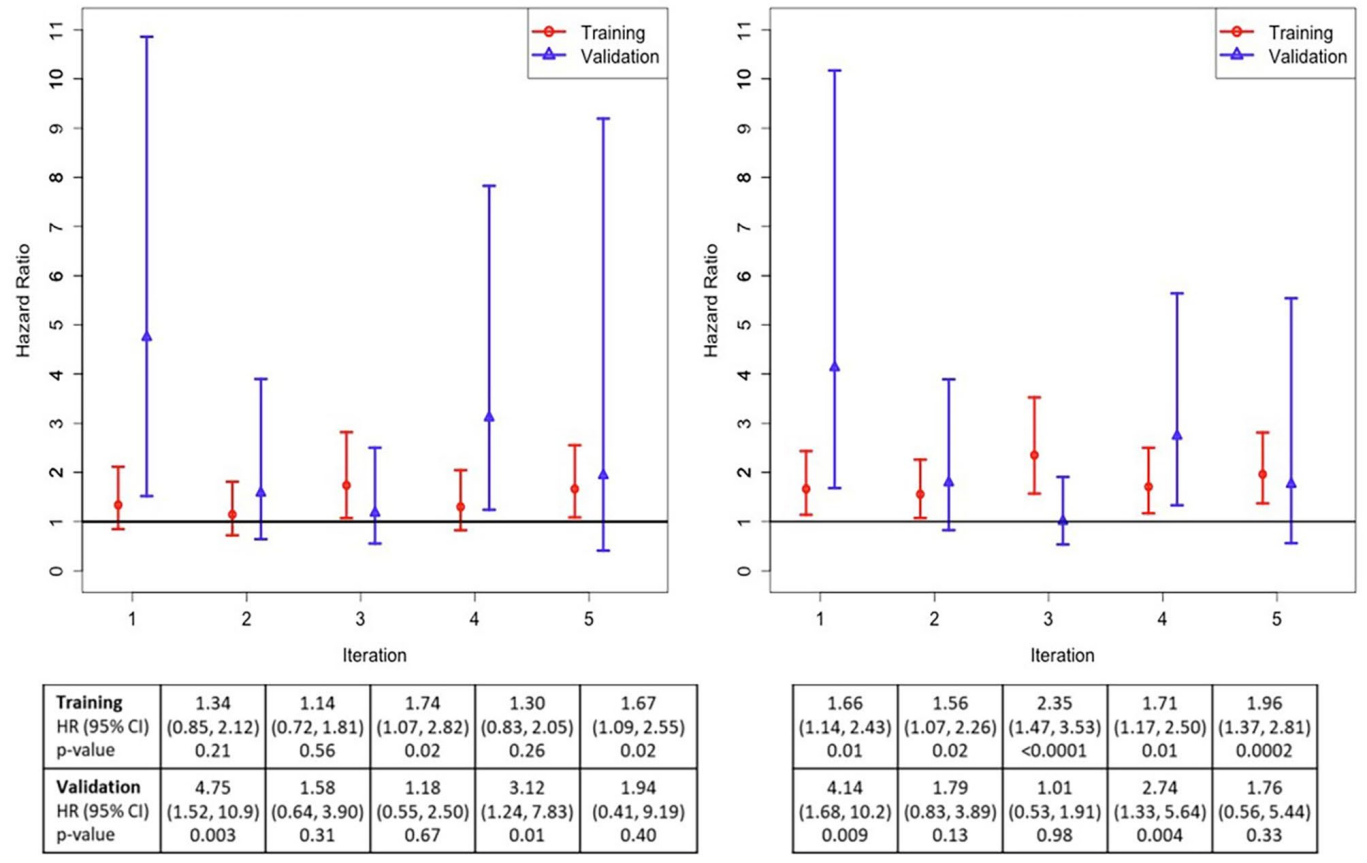
Random forest classification of subjects into molecular subtypes, based on the risk of experiencing the composite outcome of lung transplant or death (**A**), and the composite outcome of ≥ 10% absolute decline in FVC % predicted, lung transplant, or death (**B**) in each iteration of the cross-validation procedure. For all iterations, the HR was greater than 1, indicating a greater risk of these outcomes in subtype 1 compared to subtype 2. The associated tables show the HR, 95% CI, and p-values for each iteration in the training and validation datasets. HR: hazard ratio; CI: confidence interval

The random forest classifier yielded a 5-iteration mean classifier prediction area AUC of 0.95 (sd = 0.03) to predict the molecular subtypes. To further assess the classifier’s performance to identify clinically meaningful subtypes of IPF, Cox proportional hazard models estimated the risk to experience each composite endpoint in each iteration’s training and validation datasets. Although the classification of subjects to the molecular subtypes based on each iteration’s classifier did not achieve p < 0.05 in all iterations, the point estimates of HR were in the same direction, suggesting consistent classification of subjects based on clinically meaningful outcomes using the molecular data (Fig. 4). Features selected at least 3 times in all 5 iterations included 34 proteins and 7 miRNAs (see Table 2, including the mean variable importance for each molecule), and features selected in all 5 iterations included 4 proteins (BARK1, IF4G2, NDP kinase B, UFC1) and 1 miRNA (miR-744-5p).

**Table 2:** Molecules selected as classifiers of the molecular subtypes of IPF in at least 3 iterations

Molecule	Number of iterations in which molecule was selected	Variable importance across iterations where selected Mean (standard deviation)
GRB2 adapter protein	4	5.96 (1.47)
PKC-A	4	4.92 (2.16)
BCL2-like 1 protein	3	3.93 (3.26)
Sorting nexin 4	4	3.86 (1.67)
DUS3	3	3.29 (1.34)
IF4G2*	5	3.28 (2.29)
NDP kinase B*	5	3.25 (2.54)
GSK-3 alpha/beta	4	3.20 (1.42)
Cyclophilin F	3	2.97 (0.84)
DLRB1	3	2.96 (3.23)
FER	3	2.95 (2.06)
UFC1*	5	2.85 (0.91)
Caspase-3	4	2.70 (1.46)
14-3-3	3	2.66 (0.38)
PKB beta	3	2.41 (3.05)
Sphingosine kinase 1	4	2.26 (2.06)
PDE5A	4	2.19 (1.51)
HSP 60	3	2.14 (1.78)
Cofilin-1	3	2.02 (1.13)
SBDS	4	1.96 (0.62)
SMAD2	3	1.70 (0.73)
ERK-1	4	1.69 (0.95)
BARK1*	5	1.63 (0.78)
MAPK2	4	1.63 (0.99)
CSK	4	1.56 (1.01)
Calcineurin	4	1.50 (1.49)
PKB a/b/g	4	1.49 (0.72)
RAC1	3	1.48 (0.73)
IMB1	4	1.14 (0.69)
Aflatoxin B1 aldehyde reductase	3	1.02 (0.63)
Cytochrome P450 3A4	3	0.97 (0.80)
TEC	4	0.91 (0.46)
LYNB	3	0.83 (0.42)
XPNPEP1	3	0.73 (0.43)
hsa-miR-744-5p*	5	2.17 (1.33)
hsa-miR-199a-5p	3	1.94 (0.07)
hsa-miR-126-5p	3	1.61 (0.65)
hsa-miR-107	3	1.46 (0.38)
hsa-miR-3074-5p	3	1.21 (0.15)
hsa-miR-451a	3	1.15 (0.30)
hsa-miR-24-3p	3	1.10 (0.11)

*Indicates a molecule selected in all 5 classifier iterations.

The larger the variable importance score, the more relevant the variable is to the prediction.

### Coordinated alteration of biological pathways in the molecular subtypes

Linear regression identified 232 proteins (Additional file 5: Table S6), 291 miRNAs (Additional file 4: Table S7), and no toRNAs that were significantly differentially expressed or abundant between the molecular subtypes. In the IPA analysis of differentially abundant proteins, 209 down-regulated proteins (in subtype 1 compared to subtype 2) corresponded to 69 enriched pathways (Additional file 6: Table S8). Among the 291 differentially expressed miRNAs, 142 were up-regulated, corresponding to 591 experimentally-validated target genes and 345 significantly enriched pathways, and 149 were down-regulated, corresponding to 1,313 target genes and 341 significantly enriched pathways (Additional file 7: Table S9).

**Fig. 5:**
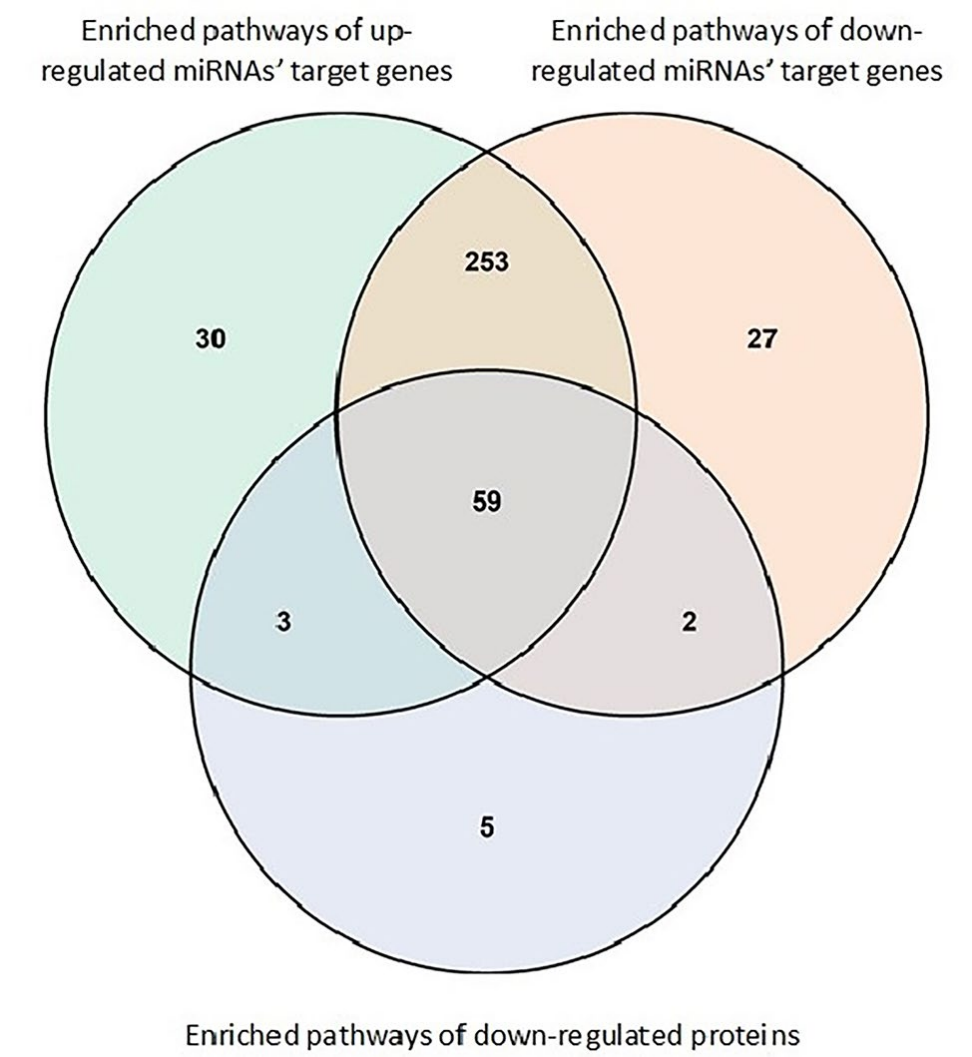
Overlap among the enriched pathways of down-regulated proteins, up-regulated target genes for miRNAs and down-regulated target genes for miRNAs.

Interestingly, there was substantial overlap in the pathway over-representation analysis for proteins and miRNA target genes (Fig. 5; and see specific pathways highlighted in Table S8 and Table S9). Pathway enrichment shared among down-regulated proteins and up-/down-regulated miRNAs included mTOR, FGF, VEGF, PDGF, ERK/MAPK signaling, NRF2-mediated oxidative stress response, and PI3K signaling in B lymphocytes. Among enriched pathways that were unique to up-regulated miRNAs, many were related to cellular or metabolic processes, such as endoplasmic reticulum stress pathway, unfolded protein response, NAD salvage pathway II, and glucose and glucose-1-phosphate degradation. Among enriched pathways that were unique to down-regulated miRNAs, many were related to immunity, including altered T and/or B cell signaling, role of RIG1-like receptors in antiviral innate immunity, and crosstalk between dendritic cells and natural killer cells.

## Discussion

In this analysis of the IPF-PRO Registry, a prospective registry of patients with IPF, we used a two-step method to harmonize multi-omics datasets and conduct unsupervised clustering based on the molecular features. This method identified two novel molecular subtypes of IPF associated with distinct clinical characteristics. Patients in subtype 1 had more severe disease at enrollment and shortened time to disease progression than patients in subtype 2, after adjusting for disease severity and use of antifibrotic treatment at baseline. The distribution of subjects into the molecular subtypes was driven by miRNA expression and protein abundance, while toRNA expression did not differ between the subtypes. Consistent with this observation, these molecular subtypes of IPF were distinct from risk groups identified using a previously described 52-gene (RNA) signature [9, 10]. A signature of 34 circulating proteins and 7 circulating miRNAs may be useful to classify patients as subtype 1 or 2. These data will be important to permit validation of the existence and clinical implications of these subtypes. A biological pathway analysis of genes encoding differentially abundant proteins or regulated by the differentially expressed miRNAs suggested a coordinated alteration of gene expression among individuals at greater risk of disease progression, including in pathways previously associated with pulmonary fibrosis.

Accurate identification of patients with IPF who are likely to experience short-term disease progression has been proposed as part of an enrichment strategy for clinical trial design [41]. Previous studies have demonstrated associations between circulating levels of protein biomarkers and IPF prognosis; most of these studies measured a limited panel of proteins (selected based on disease mechanisms), or evaluated progression-free survival without considering disease progression [42–47]. Interestingly, two independent studies found that several neoepitopes of matrix metalloprotease-degraded extracellular matrix proteins or collagen synthesis were elevated in the blood of patients with progressive IPF relative to those with stable IPF [44, 45]. Another study used an aptamer-based platform for proteomic profiling of blood in patients with IPF, and identified 9 proteins associated with IPF progression [48]. Interestingly two (carbonic anhydrase XIII and NACA) were among the 232 proteins that we identified as differentially abundant in the IPF subtypes, but while we determined that lower abundance was associated with progression, this prior study found lower abundance to be protective [48]. Similarly, the 52-gene signature has been shown to predict transplant-free survival; however, its association with disease progression has not previously been tested [9, 10]. When applied in our cohort, the high-risk group based on the 52-gene signature experienced significantly shorter transplant-free survival (as expected), but did not experience shorter progression-free survival based on a composite of ≥ 10% absolute decline in FVC % predicted, lung transplant, or death. In contrast, our molecular subtype 1 experienced shortened progression-free survival after adjusting for disease severity and antifibrotic drug use at enrollment. This suggests better resolution to predict disease progression based on multi-omics rather than gene expression (toRNA) alone. While a recent analysis suggested that longitudinal change in peripheral blood gene expression predicted a ≥ 10% decrease in FVC over follow-up [49], risk ascertainment at a single timepoint would be optimal, with the protein/miRNA classifier of IPF subtypes a candidate for further development and validation.

Integrating high-throughput data from multiple platforms remains a challenge. In this study, we initially considered three methods based on two general approaches. iCluster + and iClusterBayes include a variable selection step (i.e., lasso) followed by distillation of input matrices to a smaller set of latent variables, allowing joint clustering of samples and identification of cluster-relevant features [17, 31, 32]. Our two-step scSNF constructed a sample-similarity network (where each patient is a sample) for each omics data type and integrated these networks into a fused similarity network using a non-linear combination method [13], followed by unsupervised spectral clustering [32]. Importantly, the scSNF procedure omitted the variable selection step, limiting one source of bias.

The molecular subtypes that we identified based on integration of data from several constituents of the gene-to-protein expression pathway appear to reflect the pathobiology of IPF. Several of the proteins that were different in subtype 1 compared to 2 have been implicated in IPF pathogenesis. For example, activation of GSK-3 beta protein, which is reduced in molecular IPF subtype 1, is enhanced by TGF-beta, contributing to myofibroblast differentiation; GSK-3 beta signaling inhibition has been proposed as a treatment strategy for IPF [50]. PKB beta protein, reduced in subtype 1, has been implicated in the pathogenesis of IPF, where AKT2 knockout results in lower IL-13 and TGF-beta production by macrophages, alleviating fibrosis in animal models [51]. The MAPK/ERK pathway, of which several protein constituents were reduced in subtype 1, is activated by TGF-beta, with ERK-1/2 linked with abnormal cellular senescence [52, 53]. MAPKAPK2 (MK2) is elevated in fibroblasts and epithelial cells from patients with IPF, and its inhibition has been proposed as a treatment strategy based on pre-clinical models [54]. Interestingly, we found decreased protein abundance in the peripheral blood of persons with IPF who were at increased risk for physiologic progression, while the literature suggests that reduced quantity or activity should be protective or therapeutic. It is possible that target tissue protein quantity or activity differs from blood, but these findings may have important implications for use of blood proteins as candidate biomarkers of disease stage and/or treatment response.

Several miRNAs that have been mechanistically linked with IPF were differentially expressed in molecular IPF subtype 1 compared to 2. We identified increased expression of mir-142-5p and reduced expression of mir-130a-3p in subtype 1. Altered expression of these miRNA in macrophages (in a similar direction as we observed) has been implicated in lung and liver fibrosis via reduced STAT6 signaling; mir-142-5p targets SOCS1 (a negative regulator of STAT6 phosphorylation), and mir-130a-3p targets the PPAR-g inhibitor [16]. We found reduced expression of miR-21-3p and increased expression of miR-21-5p in molecular subtype 1. Over-expression of miR-21 has been demonstrated in the lungs of patients with IPF and in animal models of lung fibrosis, suggesting it may function via reduction of Smad7, a downstream inhibitor of TGF-beta signaling [15]. We also observed differential expression of miR-34a-5p, miR-126-5p, and miR-199a-5p in molecular subtype 1 although the direction of differential expression did not always match that expected in IPF based on published literature [55–58].

To gain additional insight into biologic differences between the molecular subtypes, canonical pathways over-representation analysis (IPA) was conducted separately for up- and down-regulated molecules in subtype 1 compared to 2. The intersection of these datasets comprised a number of pathways known to be altered in IPF (e.g., VEGF, PDGF, ERK/MAP signaling [52–54, 59]). Among non-intersecting (across proteins and miRNA) pathways, multiple innate or adaptive immunity-related pathways were over-represented among target genes of miRNA that were down-regulated in progressive IPF. Pathways that were uniquely over-represented among target genes of up-regulated miRNA in progressive IPF included a number that were related to cellular or metabolic processes. Given that miRNA often act as post-transcriptional down-regulators of gene expression, this might suggest that IPF progression is associated with increased immune responses and decreased cellular metabolism. With miRNA not extensively studied in IPF, additional research is needed to better understand these results.

Our study has several limitations. First, the aptamer-based proteomics platform we used contains a targeted list of biomarkers that is not comprehensive of all the proteins that may be found in the blood or potentially associated with pathobiology. Second, molecules measured in peripheral blood may not reflect the pathobiology of the target tissue [18, 19, 24]. Third, while this real-world registry followed participants to death or transplant, we cannot exclude the possibility that detection of disease progression based on only physiologic decline was impacted by informative missingness in lung function measurements (i.e., sicker patients were less able to complete testing). Finally, although we were able to internally validate (via resampling) our classifier of the molecular subtypes, the classifier of the molecular subtypes of IPF requires further development and validation in an independent cohort.

## Conclusions

In summary, we used a well-characterized, prospective, real-world cohort of patients with IPF to identify novel endotypes of IPF by integrating peripheral blood transcriptomic (toRNA, miRNA) and proteomic information. If externally validated, the classifier of patients with IPF to molecular subtype 1 or 2 could serve as a biomarker for prognostic enrichment in clinical trials. Constituents of the classifier, or pathways enriched among progression-associated molecules, could be explored further as therapeutic targets.

A podcast discussing these data and other analyses of circulating biomarkers in the IPF-PRO Registry is available at: https://www.usscicomms.com/respiratory/Todd/IPF-PROmultiomics.

## Electronic supplementary material

Below is the link to the electronic supplementary material.

**Additional file 1: Section S1.** Process used to quantify proteins, toRNA, and miRNA and perform bioinformatics analyses.

**Additional file 2: Table S1.** Number of toRNA, miRNA and proteomics features available for modeling after filtering and pre-processing.

**Additional file 3: **
**Section S2.** Process used to develop classifier for molecular subtypes.
**Figure S1.** Methods for development of classifier for molecular subtypes.

**Additional file 4: Section S3.** Differential expression analyses.

**Additional file 5:**
**Figure S2.** Average Silhouette scores of consensus clustering for 2 to 10 clusters.
**Section S4.** Multi-omics clustering by different methods.
**Table S2.** Cluster membership consensus between scSNF and iClusterPlus or iClusterBayes.
**Table S3.** Sensitivity analysis based on different variance filters for RNA-seq data.
**Table S4.** Comparison of molecular subtypes based on scSNF with high-risk and low-risk groups based on the 52-gene signature.
**Table S5.** Agreement (based on Normalized Mutual Information [NMI]) in distribution of subjects to molecular subtypes using a single molecule type compared to distribution using the scSNF fused multi-omics dataset.
**Table S6.** Differentially abundant proteins by molecular subtype.
**Table S7.** Differentially expressed miRNAs by molecular subtype.

**Additional file 6: Table S8.** Canonical pathways that were significantly enriched (FDR p-value < 0.05) for genes associated with down-regulated proteins.

**Additional file 7: Table S9.** Canonical pathways that were significantly enriched (FDR p-value < 0.05) for the experimentally-validated target genes of the 591 up-regulated and 1313 down-regulated miRNAs.

## Data Availability

The datasets analyzed during the current study are not publicly available, but are available from the corresponding author on reasonable request.

## Abbreviations

CPI: Composite physiologic index
DLco: Diffusion capacity of the lung for carbon monoxide
ECM: Extracellular matrix
FDR: False discovery rate
FEV1: Forced expiratory volume in the first second
FVC: Forced vital capacity
HR: Hazard ratio
IPF: Idiopathic pulmonary fibrosis
IPF: Ingenuity Pathway Analysis
MI: Myocardial infarction
miRNA: MicroRNA
NMI: Normalized Mutual Information
scSNF: Spectral clustering Similarity Network Fusion
toRNA: totalRNA
