# Supplementary material for: Integrative multi-omics analysis reveals novel idiopathic pulmonary fibrosis endotypes associated with disease progression
Source: Respir Res. 2023 May 31;24:141. doi: 10.1186/s12931-023-02435-0 (PMC10283254; doi:10.1186/s12931-023-02435-0)
Supplement: Supplementary file 1 — Additional file 1: Section S1. Process used to quantify proteins, toRNA, and miRNA and perform bioinformatics analyses. [file 12931_2023_2435_MOESM1_ESM.docx]

**Integrative Multi-Omics Analysis Reveals Novel Idiopathic Pulmonary Fibrosis Endotypes Associated with Disease Progression (****Ruan P et al)**

**Additional files**

**Additional file 1: Section S1. Process used to quantify proteins, toRNA, and miRNA and perform bioinformatics analyses.**

Proteins in plasma samples were assayed using an aptamer-based platform encompassing 1305 aptamers (SOMAscan, SOMALogic Inc., Boulder, CO) reported in relative fluorescent units (RFU). MicroRNA (miRNA) was isolated from plasma using the miRNeasy Serum/Plasma Advanced Kit (Qiagen). Libraries were prepared using the QIAseq miRNA Library Kit (Qiagen), starting with 5µL eluate for each sample. Total RNA [toRNA]was extracted from whole blood using the QIAsymphony PAXgene Blood RNA Kit (Qiagen). Libraries were prepared using TruSeq® Stranded Total RNA Library Prep Globin (Illumina) following the manufacturer's protocol, starting with 200ng toRNA. Quality of miRNA and toRNA libraries was determined using the High Sensitivity DNA Kit and the Agilent DNA 1000 Kit (Agilent Technologies), respectively, on the 2100 Bioanalyzer Instrument (Agilent Technologies). Library quantification was performed using Quant-iT™ PicoGreen dsDNA Assay Kit (Invitrogen) on a ClarioStar microplate reader (BMG Labtech). Low quality samples (miRNA library <8nM, toRNA library <6nM) were dismissed. Sequencing of miRNA and toRNA was performed on the Illumina HiSeq4000 according to the manufacturer’s instructions.

Total RNA reads were aligned to the human genome (GRCh38 primary assembly) using STAR v2.5.2a [1] with the Ensembl 84 reference genome (<http://www.ensembl.org>). FastQC v0.11.2 (<http://www.bioinformatics.babraham.ac.uk/projects/fastqc/>) was used to check read quality and RNASeQC v1.1.8 [2] was run to assess alignment quality. Duplicate reads were identified by computing duplication rates using bamUtil v1.0.11 and the dupRadar v1.4 Bioconductor R package for assessment [3]. Gene expression was quantified using Cufflinks v2.2.1[4] and feature counts [5]. Read counts were imported to R for downstream analysis.

miRNA reads were trimmed and UMIs were handled by a combination of cutadapt v1.18 [6] and a custom bash script to extract UMIs. Ribosomal (rRNA) and transfer (tRNA) RNA were removed with Bowtie2 v2.3.4.3 [7] before the filtered reads were mapped to the human genome (GRCh38 primary assembly) using the STAR aligner v2.7.0c [1]. Read counts were computed via the feature counts software package [5]. FastQC v0.11.8 (<http://www.bioinformatics.babraham.ac.uk/projects/fastqc/>) and MultiQC v1.10.1 [8] were used to assess sequencing quality at different stages (pre-trimming, after UMI trimming and based on the filtered reads). After removing rRNA/tRNA reads, cDNA, ncRNA, and piRNA were annotated before analyzing the biotype distribution using a custom R script. The analyses were based on Ensembl v86, piRBase v2.0 [9,10], GtRNAdb for GRCh38 [11,12], and miRbase v22 [13-18].

Next, data were filtered and pre-processed. toRNA and miRNA features with median cpm <1 were excluded. For toRNA and miRNA data, the count-based data normalization factors used to scale the samples based on the raw library sizes were calculated using the weighted trimmed mean of M-values (TMM) method using the default parameters of edgeR’s calcNormFactors function [19]. log2 counts per million along with associated weights for each observation were calculated using limma’s voom function [20]. toRNA features were further filtered to include only the 10% most variable features. Proteomics features below the limit of quantification were removed. Proteomics data were Log2 transformed, similar to the TMM normalization of count-based toRNA and miRNA data. Finally, data for each molecule were Box-Cox transformed and scaled to ensure normality.

**References**

1. Dobin A, Davis CA, Schlesinger F, Drenkow J, Zaleski C, Jha s, et al. STAR: ultrafast universal RNA-seq aligner. Bioinformatics. 2013;29(1):15–21.
2. DeLuca DS, Levin JZ, Sivachenko A, Fennell T, Nazaire M, Williams C, et al. RNA-SeQC: RNA-seq metrics for quality control and process optimization. Bioinformatics. 2012;28(11):1530–1532.
3. Sayols S, Scherzinger D, Klein H. dupRadar: a Bioconductor package for the assessment of PCR artifacts in RNA-Seq data. BMC Bioinformatics. 2016;17(1):428.
4. Trapnell C, Hendrickson DG, Sauvageau M, Goff L, Rinn JL, Pachter L. Differential analysis of gene regulation at transcript resolution with RNA-seq. Nat Biotechnol. 2013;31(1):46–53.
5. Liao Y, Smyth GK, Shi W. featureCounts: an efficient general purpose program for assigning sequence reads to genomic features. Bioinformatics. 2014;30:923–930.
6. Martin M. Cutadapt removes adapter sequences from high-throughput sequencing reads. EMBnet.journal. 2011;17.1:10-12.
7. Langmead B, Salzberg S. Fast gapped-read alignment with Bowtie 2. Nat Methods. 2012;9(4):357–359.
8. Ewels P, Magnusson M, Lundin S, Käller M. MultiQC: Summarize analysis results for multiple tools and samples in a single report. Bioinformatics. 2016;32(19):3047–3048.
9. Wang J, Zhang P, Lu Y, Li Y, Zheng Y, Kan Y, et al. piRBase: a comprehensive database of piRNA sequences. Nucleic Acids Res. 2019;47(D1):D175–D180.
10. Zhang P, Si X, Skogerbø G, Wang J, Cui D, Li Y, et al. piRBase: a web resource assisting piRNA functional study. Database (Oxford). 2014;2014:bau110.
11. Chan PP, Lowe TM. GtRNAdb 2.0: an expanded database of transfer RNA genes identified in complete and draft genomes. Nucleic Acids Res. 2016;44(D1):D184–D189.
12. Chan PP, Lowe TM. GtRNAdb: A database of transfer RNA genes detected in genomic sequence. Nucleic Acids Res. 2009;37(Database issue):D93-7.
13. Kozomara A, Birgaoanu M, Griffiths-Jones S. miRBase: from microRNA sequences to function. Nucleic Acids Res. 2019;47(D1):D155–D162.
14. Kozomara A, Griffiths-Jones S. miRBase: annotating high confidence microRNAs using deep sequencing data. Nucleic Acids Res. 2014;42(Database issue):D68–D73.
15. Kozomara A, Griffiths-Jones S. miRBase: integrating microRNA annotation and deep-sequencing data. Nucleic Acids Res. 2011;39(Database issue):D152–D157.
16. Griffiths-Jones S, Saini HK, van Dongen S, Enright AJ. miRBase: tools for microRNA genomics. Nucleic Acids Res. 2008;36(Database issue):D154–D158.
17. Griffiths-Jones S, Grocock RJ, van Dongen S, Bateman A, Enright AJ. miRBase: microRNA sequences, targets and gene nomenclature. Nucleic Acids Res. 2006;34(Database issue):D140–D144.
18. Griffiths‐Jones S. The microRNA Registry. Nucleic Acids Res. 2004;32(Database issue):D109–D111.
19. Robinson MD, McCarthy DJ, Smyth GK. edgeR: a Bioconductor package for differential expression analysis of digital gene expression data. Bioinformatics. 2010;26(1):139–140.
20. Law CW, Chen Y, Shi W, Smyth G Voom: precision weights unlock linear model analysis tools for RNA-seq read counts. Genome Biol. 2014;15(2):R29.
