# Supplementary material for: Integrative multi-omics analysis reveals novel idiopathic pulmonary fibrosis endotypes associated with disease progression
Source: Respir Res. 2023 May 31;24:141. doi: 10.1186/s12931-023-02435-0 (PMC10283254; doi:10.1186/s12931-023-02435-0)
Supplement: Supplementary file 2 — Additional file 2: Table S1. Number of toRNA, miRNA and proteomics features available for modeling after filtering and pre-processing. [file 12931_2023_2435_MOESM2_ESM.docx]

**Additional file 2: Table S1. Number of toRNA, miRNA and proteomics features available for modeling after filtering and pre-processing.**

|  | toRNA | miRNA | Proteomics |
| --- | --- | --- | --- |
| Total number of features measured | 60,675 | 2576 | 1305 |
| N (%) features excluded | 59,184 (97.5%) | 2104 (81.7%) | 0 (0%) |
| N (%) features analyzed | 1491 (2.5%) | 472 (18.3%) | 1305 (100%) |
