# Supplementary material for: Integrative multi-omics analysis reveals novel idiopathic pulmonary fibrosis endotypes associated with disease progression
Source: Respir Res. 2023 May 31;24:141. doi: 10.1186/s12931-023-02435-0 (PMC10283254; doi:10.1186/s12931-023-02435-0)
Supplement: Supplementary file 3 — Additional file 3: Section S2. Process used to develop classifier for molecular subtypes. Figure S1. Methods for development of classifier for molecular subtypes. [file 12931_2023_2435_MOESM3_ESM.docx]

**Additional file 3: Section S2. Process used to develop classifier for molecular subtypes.**

The random forest classifier was generated using a 5-fold cross validation strategy (**Figure S1**). First, the complete set of subjects was divided into 5 subsets (folds) of similar size by enrolling site. A site was randomly selected and its subjects set as fold 1. If the number of subjects in fold 1 was not greater than 232/5 = 46.4, subjects from another randomly selected site were added until the target N was reached. Folds 2, 3 and 4 were created by the same process. Fold 5 comprised subjects not included in folds 1-4. Next, in each of 5 iterations, a different fold was used as a validation set and the remaining folds were used for learning via the following strategy: (1) identify and retain the top 5% most significantly differentially expressed (DE) features between the subtypes for each type of molecule; (2) train a random forest model using the retained features, and calculate the variable importance score measuring each feature’s ability to distinguish the subtypes; (3) select markers with above-mean importance score to build a classifier; and (4) evaluate the performance of each classifier (developed on each of the 4 folds) to predict the subtypes in the corresponding validation fold. Performance measures included (1) mean classifier accuracy, the area under the receiver operating characteristic curve (AUC) for performance of each iteration’s classifier to predict the original subtypes in the iteration’s validation set, averaged across 5 iterations; and (2) a Cox proportional hazards regression model fitted to the training and validation fold data for each iteration, which determined the association of the training fold classifier-based subtypes with the composite outcomes. A final random forest classifier was constructed using the features selected at least 3 times among 5 iterations, and a mini-classifier was constructed using the intersection of the features in all 5 iterations.


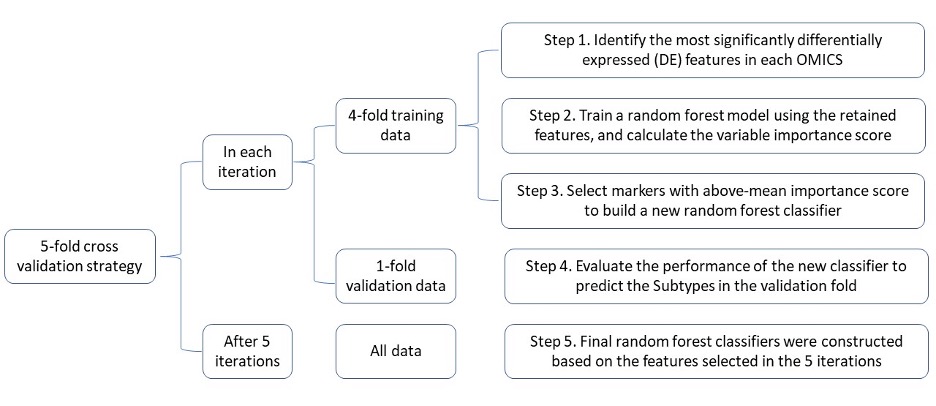
**Figure S1. Methods for development of classifier for molecular subtypes.**
