# Supplementary material for: Integrative multi-omics analysis reveals novel idiopathic pulmonary fibrosis endotypes associated with disease progression
Source: Respir Res. 2023 May 31;24:141. doi: 10.1186/s12931-023-02435-0 (PMC10283254; doi:10.1186/s12931-023-02435-0)
Supplement: Supplementary file 4 — Additional file 4: Section S3. Differential expression analyses. [file 12931_2023_2435_MOESM4_ESM.docx]

**Additional file 4: Section S3. Differential expression analyses.**

For each molecule type, differential expression analyses comparing the molecular subtypes were conducted following best practice as described in the User Guide for the Bioconductor limma package [1]. To identify differentially expressed or abundant features, linear models were fit using the lmFit function of the limma package, with fold changes and p-values derived using the contrastsfit and eBayes functions, respectively. P-values were false discovery rate (FDR)-adjusted following the Benjamini Hochberg procedure [2]. Criteria to consider a protein or gene as differentially expressed or abundant included FDR p <0.05 and |log_2_(fold change (FC))| >0.585 (corresponding to a fold-change of 1.5).

For the over-representation analysis of genes, the genes in the Ingenuity Pathway Analysis (IPA) knowledge base were used as the reference data set. For the miRNAs, experimentally-validated downstream target genes regulated by the miRNAs were determined using IPA, followed by a pathway analysis of these target genes. For the proteins, all proteins measured in the aptamer panel were used as the reference data set. When a protein aptamer mapped to multiple genes (n=17 aptamers) or when multiple aptamers mapped to a single gene (n=2 aptamers), it was excluded from the IPA analysis to minimize overrepresentation of these features.

**References**

1. Ritchie ME, Phipson B, Wu D, Hu Y, Law CW, Shi W, et al. limma powers differential expression analyses for RNA-sequencing and microarray studies. Nucleic Acids Res. 2015;43(7):e47.
2. Benjamini Y, Hochberg Y. Controlling the false discovery rate: a practical and powerful approach to multiple hypothesis testing. J R Statist Soc. 1995;57(1):289–300.
