# Supplementary material for: Integrative multi-omics analysis reveals novel idiopathic pulmonary fibrosis endotypes associated with disease progression
Source: Respir Res. 2023 May 31;24:141. doi: 10.1186/s12931-023-02435-0 (PMC10283254; doi:10.1186/s12931-023-02435-0)
Supplement: Supplementary file 5 — Additional file 5: Figure S2. Average Silhouette scores of consensus clustering for 2 to 10 clusters. Section S4. Multi-omics clustering by different methods. Table S2. Cluster membership consensus between scSNF and iClusterPlus or iClusterBayes. Table S3. Sensitivity analysis based on different variance filters for RNA-seq data. Table S4. Comparison of molecular subtypes based on scSNF with high-risk and low-risk groups based on the 52-gene signature. Table S5. Agreement (based on Normalized Mutual Information [NMI]) in distribution of subjects to molecular subtypes using a single molecule type compared to distribution using the scSNF fused multi-omics dataset. Table S6. Differentially abundant proteins by molecular subtype. Table S7. Differentially expressed miRNAs by molecular subtype. [file 12931_2023_2435_MOESM5_ESM.docx]

**Additional file 5: Figure S2.** **Average** **Silhouette scores of consensus clustering for 2 to 10 clusters.**


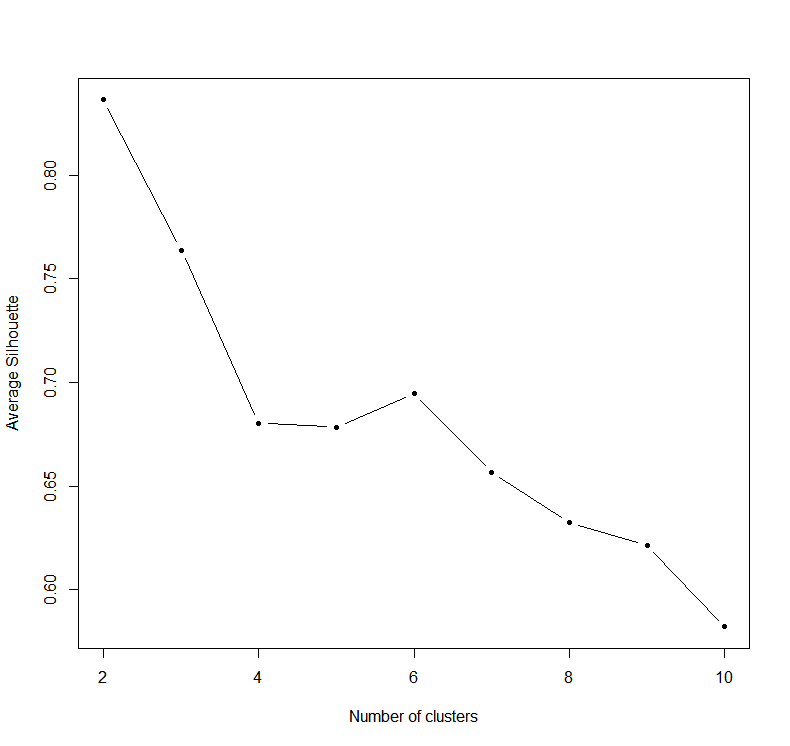


**Section S4. Multi-omics clustering by different methods.**

In addition to spectral clustering Similarity Network Fusion (scSNF), the clustering methods iCluster+ [1] and iClusterBayes [2] were assessed. iCluster+ considered Bayesian information criterion (BIC) and deviance ratio and determined the optimal number of clusters as 6, while iClusterBayes determined the optimal number of clusters as 3 using the same benchmark measures. As shown in **Table S2**, the two scSNF clusters (C1 and C2) largely overlapped the 6 iCluster+ clusters (A1-A6) and the 3 iCluster Bayes clusters (B1-B3), where C1 corresponded to B3, C2 corresponded to B1, and B2 was split between C1 and C2.

**References**

1. Mo Q, Wang S, Seshan VE, Olshen AB, Schultz N, Sander C, et al. Pattern discovery and cancer gene identification in integrated cancer genomic data. PNAS. 2013;110:4245–4250.
2. Mo Q, Shen R, Guo C, Vannucci M, Chan KS, Hilsenbeck SG. A fully Bayesian latent variable model for integrative clustering analysis of multi-type omics data. Biostatistics. 2018;19(1):71–86.

**Table S2**. **Cluster membership consensus between scSNF and iClusterPlus or iClusterBayes.**

| N subjects | | **iClusterPlus** | | | | | | | |
| --- | --- | --- | --- | --- | --- | --- | --- | --- | --- |
|  | Cluster | **A1** | **A2** | **A3** | | **A4** | **A5** | | **A6** |
| **scSNF** | **C1** | 3 | 37 | 37 | | 5 | 19 | | 4 |
|  | **C2** | 35 | 1 | 3 | | 38 | 18 | | 32 |
|  |  | **iClusterBayes** | | | | | | | |
|  | Cluster | **B1** | | | **B2** | | | **B3** | |
| **scSNF** | **C1** | 8 | | | 25 | | | 72 | |
|  | **C2** | 62 | | | 51 | | | 14 | |

**Table S3. Sensitivity analysis based on different variance filters for RNA-seq data**. The scSNF clusters based on the top 10% RNA variance filter (C1, C2) overlap the clusters based on the top 50% (M1, M2) and on 100% (N1, N2).

| **N Subjects** | | **Consensus SNF using top 50% RNA-seq (n=7455 features)** | |
| --- | --- | --- | --- |
|  | **Cluster** | **M1** | **M2** |
| scSNF **using top 10% total RNA (n=1491 features)** | **C1** | 98 | 7 |
|  | **C2** | 4 | 123 |
|  |  | **Consensus SNF using 100% RNA-seq (n=14,911 features)** | |
|  | **Cluster** | **N1** | **N2** |
| scSNF **using top 10% total RNA (n=1491 features)** | **C1** | 97 | 8 |
|  | **C2** | 5 | 122 |

**Table S4**. **Comparison of molecular subtypes based on scSNF with high-risk and low-risk groups based on the 52-gene signature.**

| **N Subjects** | | **52-gene signature** | |
| --- | --- | --- | --- |
|  | **Cluster** | **High-risk** | **Low-risk** |
| **Molecular subtype** | **Subtype 1** | 41 | 64 |
|  | **Subtype 2** | 44 | 83 |

**Table S5**. **Agreement (based on Normalized Mutual Information [NMI]) in distribution of subjects to molecular subtypes using a single molecule type compared to distribution using the scSNF fused multi-omics dataset.**

|  | | **Proteins only** (NMI=0.41) | |
| --- | --- | --- | --- |
| **scSNF** | **Molecular subtype** | P1 | P2 |
|  | S1 | 90 | 15 |
|  | S2 | 18 | 109 |
|  | | **miRNAs only** (NMI=0.62) | |
| **scSNF** | **Molecular subtype** | M1 | M2 |
|  | S1 | 101 | 4 |
|  | S2 | 14 | 113 |
|  | | **toRNAs only** (NMI=0.0003) | |
| **scSNF** | **Molecular subtype** | T1 | T2 |
|  | S1 | 55 | 50 |
|  | S2 | 69 | 58 |

**Table S6. Differentially abundant proteins by molecular subtype**. A negative (-) log (fold change) indicated that subtype 1 had a lower quantity of the protein compared to subtype 2. The 34 proteins selected by the classifier are at the top and in bold, and the 4 proteins in the mini-classifier are indicated*. The remaining proteins are arranged by p-value.

| **Protein Annotation** | **Uniprot** | **Gene Symbol** | **FDR-adjusted p-value** | **p-value** | **log(Fold Change)** |
| --- | --- | --- | --- | --- | --- |
| **BARK1*** | P25098 | ADRBK1 | 6.83E-54 | 5.23E-57 | -2.33 |
| **GSK-3 alpha/beta** | P49840 P49841 | GSK3A GSK3B | 8.85E-53 | 1.57E-55 | -2.23 |
| **IF4G2*** | P78344 | EIF4G2 | 8.85E-53 | 2.03E-55 | -2.69 |
| **PDE5A** | O76074 | PDE5A | 2.43E-52 | 7.46E-55 | -2.59 |
| **NDP kinase B*** | P22392 | NME2 | 3.71E-51 | 1.42E-53 | -2.38 |
| **Sorting nexin 4** | O95219 | SNX4 | 6.99E-51 | 3.22E-53 | -2.43 |
| **DUS3** | P51452 | DUSP3 | 1.65E-50 | 9.76E-53 | -2.08 |
| **SBDS** | Q9Y3A5 | SBDS | 1.65E-50 | 1.01E-52 | -2.52 |
| **ERK-1** | P27361 | MAPK3 | 2.86E-50 | 2.54E-52 | -1.86 |
| **FER** | P16591 | FER | 2.86E-50 | 2.63E-52 | -2.87 |
| **UFC1*** | Q9Y3C8 | UFC1 | 4.16E-50 | 4.14E-52 | -1.32 |
| **PKB beta** | P31751 | AKT2 | 7.13E-50 | 7.65E-52 | -1.92 |
| **MAPK2** | P49137 | MAPKAPK2 | 7.32E-50 | 8.42E-52 | -2.36 |
| **SMAD2** | Q15796 | SMAD2 | 1.08E-49 | 1.32E-51 | -2.42 |
| **PKC-A** | P17252 | PRKCA | 1.39E-49 | 1.82E-51 | -2.83 |
| **Caspase-3** | P42574 | CASP3 | 6.54E-49 | 9.53E-51 | -2.65 |
| **TEC** | P42680 | TEC | 1.67E-48 | 2.56E-50 | -1.96 |
| **GRB2 adapter protein** | P62993 | GRB2 | 2.22E-48 | 3.57E-50 | -2.29 |
| **Sphingosine kinase 1** | Q9NYA1 | SPHK1 | 4.68E-48 | 8.26E-50 | -2.71 |
| **LYNB** | P07948 | LYN | 5.95E-48 | 1.10E-49 | -2.29 |
| **BCL2-like 1 protein** | Q07817 | BCL2L1 | 9.52E-48 | 2.04E-49 | -1.17 |
| **HSP 60** | P10809 | HSPD1 | 1.15E-47 | 2.56E-49 | -2.46 |
| **RAC1** | P63000 | RAC1 | 1.25E-47 | 2.87E-49 | -2.21 |
| **Cofilin-1** | P23528 | CFL1 | 3.16E-47 | 7.51E-49 | -1.44 |
| **CSK** | P41240 | CSK | 4.53E-47 | 1.15E-48 | -3.02 |
| **PKB a/b/g** | P31749 P31751 Q9Y243 | AKT1 AKT2 AKT3 | 8.16E-47 | 2.13E-48 | -1.86 |
| **Aflatoxin B1 aldehyde reductase** | O43488 | AKR7A2 | 1.06E-46 | 3.00E-48 | -2.55 |
| **14-3-3** | P31946 P62258 P619 | YWHAB YWHAE YWHAG YWHAH YWHAQ YWHAZ SFN | 1.37E-45 | 4.39E-47 | -2.15 |
| **Cytochrome P450 3A4** | P08684 | CYP3A4 | 2.90E-45 | 9.99E-47 | -1.42 |
| **Cyclophilin F** | P30405 | PPIF | 3.61E-45 | 1.30E-46 | -2.97 |
| **Calcineurin** | Q08209 P63098 | PPP3CA PPP3R1 | 3.83E-45 | 1.41E-46 | -1.95 |
| **DLRB1** | Q9NP97 | DYNLRB1 | 1.31E-43 | 5.51E-45 | -2.10 |
| **IMB1** | Q14974 | KPNB1 | 1.07E-41 | 5.75E-43 | -2.27 |
| **XPNPEP1** | Q9NQW7 | XPNPEP1 | 7.63E-38 | 5.50E-39 | -1.64 |
| AIP | O00170 | AIP | 1.80E-50 | 1.24E-52 | -1.76 |
| DEAD-box protein 19B | Q9UMR2 | DDX19B | 2.86E-50 | 2.40E-52 | -1.80 |
| ERAB | Q99714 | HSD17B10 | 6.16E-49 | 8.49E-51 | -2.31 |
| SHC1 | P29353 | SHC1 | 4.48E-48 | 7.56E-50 | -1.53 |
| Carbonic anhydrase XIII | Q8N1Q1 | CA13 | 8.42E-48 | 1.67E-49 | -2.65 |
| LYN | P07948 | LYN | 8.42E-48 | 1.68E-49 | -2.47 |
| AMPK a2b2g1 | P54646 O43741 P54619 | PRKAA2 PRKAB2 PRKAG1 | 8.69E-48 | 1.80E-49 | -1.80 |
| M2-PK | P14618 | PKM2 | 4.46E-47 | 1.09E-48 | -2.55 |
| SGTA | O43765 | SGTA | 9.16E-47 | 2.46E-48 | -2.06 |
| PDPK1 | O15530 | PDPK1 | 1.01E-46 | 2.79E-48 | -2.39 |
| BTK | Q06187 | BTK | 1.46E-46 | 4.25E-48 | -3.20 |
| NCC27 | O00299 | CLIC1 | 2.68E-46 | 8.02E-48 | -2.40 |
| TCTP | P13693 | TPT1 | 3.90E-46 | 1.19E-47 | -2.05 |
| PKC-B-II | P05771 | PRKCB | 8.12E-46 | 2.55E-47 | -3.08 |
| 14-3-3 protein beta/alpha | P31946 | YWHAB | 2.61E-45 | 8.59E-47 | -2.07 |
| METAP1 | P53582 | METAP1 | 2.68E-45 | 9.03E-47 | -2.39 |
| Flt-3 | P36888 | FLT3 | 3.18E-45 | 1.12E-46 | -1.63 |
| PPID | Q08752 | PPID | 6.67E-45 | 2.50E-46 | -3.16 |
| PPIE | Q9UNP9 | PPIE | 8.71E-45 | 3.34E-46 | -1.77 |
| SMAD3 | P84022 | SMAD3 | 2.02E-44 | 7.91E-46 | -1.47 |
| MAPK14 | Q16539 | MAPK14 | 2.82E-44 | 1.13E-45 | -1.62 |
| UFM1 | P61960 | UFM1 | 3.31E-44 | 1.35E-45 | -1.83 |
| Cyclophilin A | P62937 | PPIA | 4.74E-44 | 1.96E-45 | -1.10 |
| DRG-1 | Q9NP79 | VTA1 | 4.52E-43 | 1.94E-44 | -2.12 |
| SRCN1 | P12931 | SRC | 5.02E-43 | 2.19E-44 | -2.66 |
| AMPM2 | P50579 | METAP2 | 5.20E-43 | 2.31E-44 | -1.39 |
| Transketolase | P29401 | TKT | 1.03E-42 | 4.65E-44 | -1.44 |
| Calpain I | P07384 P04632 | CAPN1 CAPNS1 | 1.44E-42 | 6.64E-44 | -1.42 |
| Alpha enolase | P06733 | ENO1 | 1.59E-42 | 7.42E-44 | -1.67 |
| HSP 27 | P04792 | HSPB1 | 1.78E-42 | 8.48E-44 | -2.30 |
| aldolase A | P04075 | ALDOA | 2.78E-42 | 1.34E-43 | -0.93 |
| BAD | Q92934 | BAD | 2.78E-42 | 1.36E-43 | -1.70 |
| MAPKAPK3 | Q16644 | MAPKAPK3 | 2.80E-42 | 1.40E-43 | -1.81 |
| CK2-A1:B | P68400 P67870 | CSNK2A1 CSNK2B | 6.04E-42 | 3.06E-43 | -1.81 |
| VAV | P15498 | VAV1 | 6.13E-42 | 3.15E-43 | -2.35 |
| eIF-4H | Q15056 | EIF4H | 6.89E-42 | 3.59E-43 | -2.53 |
| HSP 90b | P08238 | HSP90AB1 | 7.94E-42 | 4.20E-43 | -1.09 |
| STAT3 | P40763 | STAT3 | 1.44E-41 | 7.81E-43 | -1.97 |
| GAPDH, liver | P04406 | GAPDH | 1.89E-41 | 1.04E-42 | -1.65 |
| HSP 40 | P25685 | DNAJB1 | 3.50E-41 | 1.96E-42 | -1.77 |
| ING1 | Q9UK53 | ING1 | 3.75E-41 | 2.13E-42 | -1.96 |
| Triosephosphate isomerase | P60174 | TPI1 | 1.68E-40 | 9.65E-42 | -1.76 |
| 14-3-3 protein zeta/delta | P63104 | YWHAZ | 2.01E-40 | 1.17E-41 | -1.55 |
| GPVI | Q9HCN6 | GP6 | 2.71E-40 | 1.60E-41 | -1.66 |
| eIF-5A-1 | P63241 | EIF5A | 6.95E-40 | 4.15E-41 | -1.32 |
| NACA | Q13765 | NACA | 7.88E-40 | 4.77E-41 | -1.58 |
| RPS6KA3 | P51812 | RPS6KA3 | 8.37E-40 | 5.13E-41 | -1.60 |
| IL-2 sRg | P31785 | IL2RG | 1.06E-39 | 6.56E-41 | -1.60 |
| 6-Phosphogluconate dehydrogenase | P52209 | PGD | 2.07E-39 | 1.30E-40 | -2.12 |
| MK08 | P45983 | MAPK8 | 3.59E-39 | 2.31E-40 | -1.59 |
| PTP-1C | P29350 | PTPN6 | 3.59E-39 | 2.31E-40 | -1.84 |
| CDK8/cyclin C | P49336 P24863 | CDK8 CCNC | 5.79E-39 | 3.77E-40 | -0.82 |
| Transgelin-2 | P37802 | TAGLN2 | 6.29E-39 | 4.14E-40 | -1.34 |
| PAK6 | Q9NQU5 | PAK6 | 8.24E-39 | 5.50E-40 | -2.63 |
| FYN | P06241 | FYN | 1.03E-38 | 6.91E-40 | -2.06 |
| PHI | P06744 | GPI | 2.36E-38 | 1.61E-39 | -1.16 |
| STAT1 | P42224 | STAT1 | 2.79E-38 | 1.92E-39 | -1.87 |
| HSP 90a/b | P07900 P08238 | HSP90AA1 HSP90AB1 | 2.87E-38 | 2.00E-39 | -1.50 |
| Tropomyosin 4 | P67936 | TPM4 | 4.04E-38 | 2.85E-39 | -2.56 |
| CONA1 | Q86Y22 | COL23A1 | 7.22E-38 | 5.14E-39 | -1.30 |
| ARP19 | P56211 | ARPP19 | 9.71E-38 | 7.07E-39 | -1.76 |
| CPNE1 | Q99829 | CPNE1 | 1.91E-37 | 1.41E-38 | -2.13 |
| MP2K3 | P46734 | MAP2K3 | 4.28E-37 | 3.18E-38 | -1.17 |
| SNAA | P54920 | NAPA | 7.40E-37 | 5.56E-38 | -1.70 |
| HDAC8 | Q9BY41 | HDAC8 | 1.32E-36 | 1.00E-37 | -0.62 |
| HEMK2 | Q9Y5N5 | N6AMT1 | 1.38E-36 | 1.05E-37 | -1.49 |
| YES | P07947 | YES1 | 2.21E-36 | 1.71E-37 | -0.81 |
| FGF-16 | O43320 | FGF16 | 2.43E-36 | 1.90E-37 | -1.52 |
| SUMO3 | P55854 | SUMO3 | 2.90E-36 | 2.29E-37 | -1.53 |
| PRKACA | P17612 | PRKACA | 3.37E-36 | 2.68E-37 | -1.84 |
| UBE2N | P61088 | UBE2N | 1.52E-35 | 1.22E-36 | -1.67 |
| KPCT | Q04759 | PRKCQ | 1.62E-35 | 1.31E-36 | -1.42 |
| SHP-2 | Q06124 | PTPN11 | 2.06E-35 | 1.69E-36 | -1.21 |
| a-Synuclein | P37840 | SNCA | 3.97E-35 | 3.29E-36 | -1.24 |
| RAC3 | P60763 | RAC3 | 4.50E-35 | 3.76E-36 | -1.19 |
| JAM-C | Q9BX67 | JAM3 | 6.46E-35 | 5.44E-36 | -1.11 |
| PA2G4 | Q9UQ80 | PA2G4 | 9.03E-35 | 7.68E-36 | -1.70 |
| Protein disulfide isomerase A3 | P30101 | PDIA3 | 2.94E-34 | 2.52E-35 | -1.14 |
| CAMK2A | Q9UQM7 | CAMK2A | 3.74E-34 | 3.24E-35 | -1.36 |
| CAMK2D | Q13557 | CAMK2D | 4.14E-34 | 3.61E-35 | -1.60 |
| MK01 | P28482 | MAPK1 | 8.66E-34 | 7.63E-35 | -1.81 |
| DHH | O43323 | DHH | 1.10E-33 | 9.75E-35 | -1.17 |
| PDE11 | Q9HCR9 | PDE11A | 1.24E-33 | 1.11E-34 | -1.06 |
| Stress-induced-phosphoprotein 1 | P31948 | STIP1 | 2.49E-33 | 2.25E-34 | -1.41 |
| UB2L3 | P68036 | UBE2L3 | 4.09E-33 | 3.73E-34 | -1.35 |
| BID | P55957 | BID | 4.50E-33 | 4.13E-34 | -1.19 |
| CLC1B | Q9P126 | CLEC1B | 6.66E-33 | 6.17E-34 | -1.26 |
| CK2-A2:B | P19784 P67870 | CSNK2A2 CSNK2B | 7.69E-33 | 7.19E-34 | -1.37 |
| UBC9 | P63279 | UBE2I | 9.97E-33 | 9.39E-34 | -1.46 |
| NMT1 | P30419 | NMT1 | 1.38E-32 | 1.31E-33 | -1.53 |
| AREG | P15514 | AREG | 1.48E-32 | 1.42E-33 | -1.54 |
| KREM2 | Q8NCW0 | KREMEN2 | 1.56E-32 | 1.50E-33 | -1.04 |
| EP15R | Q9UBC2 | EPS15L1 | 7.47E-32 | 7.27E-33 | -1.03 |
| PLPP | Q96GD0 | PDXP | 1.95E-31 | 1.91E-32 | -1.52 |
| Rab GDP dissociation inhibitor beta | P50395 | GDI2 | 2.10E-31 | 2.08E-32 | -1.07 |
| CAMK2B | Q13554 | CAMK2B | 1.61E-30 | 1.60E-31 | -1.47 |
| ATPO | P48047 | ATP5O | 2.01E-30 | 2.01E-31 | -1.63 |
| PAFAH beta subunit | P68402 | PAFAH1B2 | 2.73E-30 | 2.76E-31 | -0.98 |
| EDAR | Q9UNE0 | EDAR | 1.15E-29 | 1.17E-30 | -1.28 |
| CRK | P46108 | CRK | 2.57E-29 | 2.64E-30 | -1.33 |
| IL-3 Ra | P26951 | IL3RA | 3.34E-29 | 3.45E-30 | -1.20 |
| PIK3CA/PIK3R1 | P42336 P27986 | PIK3CA PIK3R1 | 3.71E-29 | 3.87E-30 | -0.95 |
| MDHC | P40925 | MDH1 | 5.46E-29 | 5.73E-30 | -0.90 |
| Peroxiredoxin-6 | P30041 | PRDX6 | 1.05E-28 | 1.11E-29 | -1.49 |
| NSF1C | Q9UNZ2 | NSFL1C | 1.26E-28 | 1.35E-29 | -1.39 |
| CHIP | Q9UNE7 | STUB1 | 2.11E-28 | 2.26E-29 | -1.27 |
| PSA6 | P60900 | PSMA6 | 2.13E-28 | 2.30E-29 | -1.11 |
| prostatic binding protein | P30086 | PEBP1 | 2.28E-28 | 2.48E-29 | -1.05 |
| RAN | P62826 | RAN | 3.03E-28 | 3.32E-29 | -1.87 |
| phosphoglycerate kinase 1 | P00558 | PGK1 | 5.51E-27 | 6.08E-28 | -1.08 |
| PGP9.5 | P09936 | UCHL1 | 1.20E-26 | 1.33E-27 | -0.94 |
| EF-1-beta | P24534 | EEF1B2 | 1.61E-26 | 1.80E-27 | -1.01 |
| AK1A1 | P14550 | AKR1A1 | 5.95E-26 | 6.70E-27 | -0.93 |
| KPCI | P41743 | PRKCI | 7.03E-26 | 7.97E-27 | -1.04 |
| COMMD7 | Q86VX2 | COMMD7 | 3.32E-25 | 3.79E-26 | -0.82 |
| SKP1 | P63208 | SKP1 | 5.37E-25 | 6.17E-26 | -0.81 |
| DBNL | Q9UJU6 | DBNL | 5.81E-25 | 6.72E-26 | -0.98 |
| FLRT1 | Q9NZU1 | FLRT1 | 6.81E-25 | 7.93E-26 | -0.83 |
| RSK-like protein kinase | O75582 | RPS6KA5 | 2.12E-24 | 2.49E-25 | -0.94 |
| Gro-b/g | P19876 P19875 | CXCL3 CXCL2 | 3.67E-24 | 4.33E-25 | -1.33 |
| Testican-1 | Q08629 | SPOCK1 | 9.29E-24 | 1.10E-24 | -0.78 |
| IFN-lambda 1 | Q8IU54 | IFNL1 | 1.53E-23 | 1.82E-24 | -0.68 |
| MFGM | Q08431 | MFGE8 | 3.22E-23 | 3.89E-24 | 1.00 |
| VEGF-C | P49767 | VEGFC | 3.35E-23 | 4.09E-24 | -0.66 |
| FGF7 | P21781 | FGF7 | 1.31E-22 | 1.62E-23 | -0.66 |
| CaMKK alpha | Q8N5S9 | CAMKK1 | 2.00E-22 | 2.48E-23 | -0.96 |
| DAPK2 | Q9UIK4 | DAPK2 | 2.27E-22 | 2.84E-23 | -0.64 |
| FGF-6 | P10767 | FGF6 | 2.17E-21 | 2.73E-22 | -0.94 |
| HTRA2 | O43464 | HTRA2 | 2.30E-21 | 2.91E-22 | -0.71 |
| PPase | Q15181 | PPA1 | 2.69E-21 | 3.43E-22 | -0.97 |
| KIF23 | Q02241 | KIF23 | 4.01E-21 | 5.13E-22 | -1.03 |
| Peroxiredoxin-1 | Q06830 | PRDX1 | 4.27E-21 | 5.50E-22 | -1.06 |
| WNK3 | Q9BYP7 | WNK3 | 4.33E-21 | 5.61E-22 | -1.04 |
| Ubiquitin+1 | P62979 | RPS27A | 4.72E-21 | 6.15E-22 | -1.04 |
| IL-6 | P05231 | IL6 | 1.32E-20 | 1.72E-21 | -0.75 |
| PKC-D | Q05655 | PRKCD | 1.45E-20 | 1.91E-21 | -0.81 |
| ULBP-3 | Q9BZM4 | ULBP3 | 1.78E-20 | 2.37E-21 | -0.79 |
| PSME1 | Q06323 | PSME1 | 2.44E-20 | 3.28E-21 | -0.80 |
| IMDH2 | P12268 | IMPDH2 | 5.97E-20 | 8.05E-21 | -0.60 |
| LDH-H 1 | P07195 | LDHB | 6.17E-20 | 8.37E-21 | -0.86 |
| TAK1-TAB1 | O43318 Q15750 | MAP3K7 TAB1 | 6.20E-20 | 8.46E-21 | -0.80 |
| Vasoactive Intestinal Peptide | P01282 | VIP | 6.65E-20 | 9.12E-21 | -0.69 |
| IMDH1 | P20839 | IMPDH1 | 8.12E-20 | 1.12E-20 | -0.92 |
| NUDC3 | Q8IVD9 | NUDCD3 | 1.82E-19 | 2.52E-20 | -0.62 |
| MDM2 | Q00987 | MDM2 | 2.24E-19 | 3.13E-20 | -0.59 |
| Nucleoside diphosphate kinase A | P15531 | NME1 | 2.25E-19 | 3.15E-20 | -0.68 |
| B7-H2 | O75144 | ICOSLG | 2.32E-19 | 3.27E-20 | -1.17 |
| hnRNP A/B | Q99729 | HNRNPAB | 5.90E-19 | 8.37E-20 | -0.97 |
| STAT6 | P42226 | STAT6 | 1.30E-18 | 1.86E-19 | -0.78 |
| NAP-2 | P02775 | PPBP | 1.43E-18 | 2.05E-19 | -1.36 |
| CTAP-III | P02775 | PPBP | 1.62E-18 | 2.33E-19 | -1.36 |
| NSE | P09104 | ENO2 | 1.87E-18 | 2.71E-19 | -0.97 |
| CDC37 | Q16543 | CDC37 | 2.55E-18 | 3.71E-19 | -0.73 |
| SE6L2 | Q6UXD5 | SEZ6L2 | 3.86E-18 | 5.65E-19 | -1.09 |
| Myokinase, human | P00568 | AK1 | 5.27E-18 | 7.75E-19 | -0.83 |
| CD47 | Q08722 | CD47 | 5.51E-18 | 8.14E-19 | -0.79 |
| HINT1 | P49773 | HINT1 | 1.56E-17 | 2.33E-18 | -0.62 |
| IL-23 R | Q5VWK5 | IL23R | 1.66E-17 | 2.49E-18 | -0.65 |
| ARI3A | Q99856 | ARID3A | 2.01E-17 | 3.03E-18 | -0.83 |
| MK12 | P53778 | MAPK12 | 3.06E-16 | 4.67E-17 | -0.71 |
| MIF | P14174 | MIF | 3.53E-16 | 5.43E-17 | -0.84 |
| PF-4 | P02776 | PF4 | 5.96E-16 | 9.22E-17 | -1.33 |
| PTP-1B | P18031 | PTPN1 | 5.96E-16 | 9.26E-17 | -0.60 |
| Azurocidin | P20160 | AZU1 | 3.00E-15 | 4.74E-16 | -0.68 |
| CSH | P0DML2 P0DML3 | CSH1 CSH2 | 3.02E-15 | 4.80E-16 | -0.73 |
| PPAC | P24666 | ACP1 | 3.22E-15 | 5.14E-16 | -1.36 |
| FGF-8A | P55075 | FGF8 | 5.40E-15 | 8.69E-16 | -0.84 |
| S100A6 | P06703 | S100A6 | 7.95E-14 | 1.34E-14 | -0.66 |
| Phosphoglycerate mutase 1 | P18669 | PGAM1 | 2.24E-13 | 3.81E-14 | -1.98 |
| Integrin a1b1 | P56199 P05556 | ITGA1 ITGB1 | 5.49E-13 | 9.64E-14 | -0.67 |
| CD40 ligand, soluble | P29965 | CD40LG | 5.62E-13 | 9.90E-14 | -0.71 |
| TARC | Q92583 | CCL17 | 8.76E-13 | 1.55E-13 | -1.06 |
| Thrombospondin-1 | P07996 | THBS1 | 2.25E-12 | 4.10E-13 | -1.07 |
| PDGF-BB | P01127 | PDGFB | 3.48E-12 | 6.43E-13 | -1.01 |
| SOD | P00441 | SOD1 | 4.85E-12 | 9.03E-13 | -0.59 |
| annexin II | P07355 | ANXA2 | 5.08E-12 | 9.53E-13 | -0.69 |
| Bcl-2 | P10415 | BCL2 | 5.92E-12 | 1.12E-12 | -0.61 |
| PDGF-AA | P04085 | PDGFA | 7.53E-12 | 1.45E-12 | -0.84 |
| FUT5 | Q11128 | FUT5 | 7.86E-12 | 1.54E-12 | 0.64 |
| RANTES | P13501 | CCL5 | 9.94E-12 | 1.97E-12 | -0.86 |
| Mesothelin | Q13421 | MSLN | 1.61E-11 | 3.29E-12 | -0.67 |
| Luteinizing hormone | P01215 P01229 | CGA LHB | 3.12E-11 | 6.38E-12 | -0.84 |
| CTGF | P29279 | CTGF | 5.32E-11 | 1.10E-11 | -0.78 |
| RS3 | P23396 | RPS3 | 7.29E-11 | 1.52E-11 | -0.98 |
| PDK1 | Q15118 | PDK1 | 1.36E-10 | 2.89E-11 | -0.76 |
| ON | P09486 | SPARC | 1.43E-10 | 3.06E-11 | -0.72 |
| RS7 | P62081 | RPS7 | 2.89E-10 | 6.35E-11 | -0.70 |
| 41 | P11171 | EPB41 | 2.02E-09 | 4.85E-10 | -0.60 |
| FGR | P09769 | FGR | 2.18E-09 | 5.24E-10 | -0.82 |
| amyloid precursor protein | P05067 | APP | 1.61E-08 | 4.05E-09 | -0.61 |
| annexin VI | P08133 | ANXA6 | 2.52E-08 | 6.61E-09 | -0.70 |
| TIMP-3 | P35625 | TIMP3 | 3.62E-07 | 1.12E-07 | -0.63 |
| GCP-2 | P80162 | CXCL6 | 7.68E-06 | 2.80E-06 | -0.61 |
| BLC | O43927 | CXCL13 | 9.29E-06 | 3.40E-06 | 0.59 |
| FTCD | O95954 | FTCD | 1.59E-05 | 6.01E-06 | 0.86 |
| SP-D | P35247 | SFTPD | 6.29E-05 | 2.64E-05 | -0.72 |
| bFGF | P09038 | FGF2 | 0.000373 | 0.000174 | -0.63 |
| IgE | P01854 | IGHE | 0.001638 | 0.00085 | 0.94 |

**Table S7. Differentially expressed miRNAs by molecular subtype.** A negative (-) log (fold change) indicated that subtype 1 had lower expression of the miRNA compared to subtype 2. The 7 miRNAs selected by the classifier are at the top and in bold and the miRNAs in the mini-classifier are indicated*. The remaining miRNAs are arranged by p-value.

| **miRNA** | **FDR-adjusted p-value** | **p-value** | **Log(fold change)** |
| --- | --- | --- | --- |
| **hsa-miR-744-5p*** | 3.47E-45 | 7.36E-48 | -1.43445 |
| **hsa-miR-126-5p** | 6.06E-42 | 2.57E-44 | -0.94446 |
| **hsa-miR-3074-5p** | 8.30E-38 | 1.07E-39 | -1.01591 |
| **hsa-miR-24-3p** | 8.30E-38 | 1.09E-39 | -1.01593 |
| **hsa-miR-199a-5p** | 1.23E-36 | 2.34E-38 | -1.50724 |
| **hsa-miR-107** | 1.42E-30 | 6.33E-32 | 1.27877 |
| **hsa-miR-451a** | 8.27E-29 | 4.73E-30 | 1.591456 |
| hsa-miR-340-5p | 2.56E-38 | 1.63E-40 | -1.13921 |
| hsa-miR-148b-3p | 6.65E-38 | 5.64E-40 | -0.7841 |
| hsa-miR-151a-3p | 8.30E-38 | 1.23E-39 | -1.2781 |
| hsa-miR-223-3p | 1.23E-36 | 2.08E-38 | -1.26911 |
| hsa-miR-146a-5p | 1.65E-35 | 3.51E-37 | -1.2322 |
| hsa-miR-28-3p | 1.85E-35 | 4.32E-37 | -1.13556 |
| hsa-miR-326 | 6.73E-35 | 1.71E-36 | -1.80519 |
| hsa-miR-126-3p | 1.13E-34 | 3.11E-36 | -0.77617 |
| hsa-miR-199b-3p | 1.52E-33 | 4.83E-35 | -1.2014 |
| hsa-miR-199a-3p | 1.52E-33 | 4.84E-35 | -1.20139 |
| hsa-miR-32-5p | 9.40E-33 | 3.19E-34 | 1.180088 |
| hsa-miR-221-3p | 2.92E-32 | 1.05E-33 | -0.9197 |
| hsa-miR-584-5p | 6.64E-32 | 2.53E-33 | -1.22819 |
| hsa-miR-628-3p | 1.76E-31 | 7.09E-33 | -1.18229 |
| hsa-miR-4732-5p | 1.02E-30 | 4.33E-32 | 1.786377 |
| hsa-miR-486-5p | 6.13E-30 | 2.99E-31 | 1.420726 |
| hsa-miR-486-3p | 6.13E-30 | 2.99E-31 | 1.420722 |
| hsa-miR-26a-1-3p | 1.02E-29 | 5.17E-31 | -1.63007 |
| hsa-miR-6852-5p | 1.38E-29 | 7.33E-31 | -1.70177 |
| hsa-miR-335-5p | 5.72E-29 | 3.15E-30 | -1.03138 |
| hsa-miR-191-3p | 1.58E-28 | 9.36E-30 | -1.23779 |
| hsa-miR-1307-3p | 2.22E-28 | 1.36E-29 | -1.09248 |
| hsa-miR-30e-3p | 3.00E-28 | 1.91E-29 | -0.9451 |
| hsa-let-7d-5p | 8.27E-28 | 5.43E-29 | 0.966532 |
| hsa-miR-4732-3p | 1.87E-27 | 1.27E-28 | 1.86526 |
| hsa-miR-1296-5p | 3.29E-27 | 2.30E-28 | -1.56668 |
| hsa-miR-3613-5p | 8.72E-27 | 6.28E-28 | 1.212948 |
| hsa-miR-532-3p | 1.42E-26 | 1.05E-27 | 1.155396 |
| hsa-miR-1301-3p | 3.91E-26 | 2.98E-27 | -1.27021 |
| hsa-miR-152-3p | 1.73E-25 | 1.40E-26 | -0.82913 |
| hsa-miR-16-5p | 1.70E-25 | 1.33E-26 | 0.972611 |
| hsa-miR-451b | 7.14E-25 | 5.90E-26 | 1.862957 |
| hsa-miR-194-5p | 9.67E-25 | 8.20E-26 | 1.334675 |
| hsa-miR-25-3p | 1.40E-24 | 1.21E-25 | 0.81148 |
| hsa-miR-335-3p | 1.96E-24 | 1.74E-25 | -1.51682 |
| hsa-miR-1294 | 2.07E-24 | 1.89E-25 | 1.409903 |
| hsa-miR-16-2-3p | 2.27E-24 | 2.12E-25 | 1.182943 |
| hsa-miR-182-5p | 2.88E-24 | 2.74E-25 | 1.416636 |
| hsa-miR-339-5p | 7.06E-24 | 7.03E-25 | -1.29069 |
| hsa-miR-30d-5p | 6.32E-24 | 6.16E-25 | -0.5921 |
| hsa-miR-185-3p | 1.31E-23 | 1.33E-24 | -1.12619 |
| hsa-miR-363-3p | 2.01E-23 | 2.09E-24 | 1.095941 |
| hsa-miR-3529-3p | 2.07E-23 | 2.20E-24 | 1.230957 |
| hsa-miR-7-5p | 2.53E-23 | 2.74E-24 | 1.225786 |
| hsa-miR-3615 | 2.73E-23 | 3.01E-24 | 0.862206 |
| hsa-miR-10b-5p | 2.85E-23 | 3.20E-24 | 1.71148 |
| hsa-miR-381-3p | 4.02E-23 | 4.60E-24 | -1.77758 |
| hsa-miR-330-3p | 5.76E-23 | 6.71E-24 | -1.34899 |
| hsa-miR-15b-5p | 6.97E-23 | 8.27E-24 | 0.821429 |
| hsa-miR-134-5p | 1.24E-22 | 1.50E-23 | -1.73064 |
| hsa-miR-15a-5p | 1.34E-22 | 1.65E-23 | 1.021189 |
| hsa-miR-654-3p | 1.61E-22 | 2.01E-23 | -1.7548 |
| hsa-miR-193a-5p | 1.67E-22 | 2.13E-23 | 1.39821 |
| hsa-miR-127-3p | 3.33E-22 | 4.38E-23 | -1.77972 |
| hsa-miR-183-5p | 3.12E-22 | 4.03E-23 | 1.487568 |
| hsa-miR-409-3p | 3.52E-22 | 4.70E-23 | -1.79825 |
| hsa-miR-29c-3p | 5.55E-22 | 7.53E-23 | 0.82353 |
| hsa-miR-4446-3p | 1.93E-21 | 2.65E-22 | -1.86246 |
| hsa-miR-375 | 2.49E-21 | 3.54E-22 | 1.870801 |
| hsa-miR-483-5p | 2.49E-21 | 3.51E-22 | 1.907235 |
| hsa-miR-223-5p | 2.75E-21 | 3.96E-22 | -0.78457 |
| hsa-miR-660-5p | 2.97E-21 | 4.35E-22 | 0.883156 |
| hsa-miR-192-5p | 4.10E-21 | 6.08E-22 | 1.155744 |
| hsa-miR-625-5p | 5.21E-21 | 7.86E-22 | -1.09277 |
| hsa-miR-625-3p | 5.21E-21 | 7.95E-22 | -1.09288 |
| hsa-miR-502-3p | 7.65E-21 | 1.20E-21 | 1.143716 |
| hsa-let-7a-5p | 5.88E-21 | 9.09E-22 | 0.889047 |
| hsa-miR-144-5p | 8.81E-21 | 1.40E-21 | 1.764241 |
| hsa-miR-361-5p | 9.24E-21 | 1.49E-21 | -0.61165 |
| hsa-miR-146b-5p | 1.18E-20 | 1.95E-21 | -0.98826 |
| hsa-miR-1180-3p | 1.40E-20 | 2.37E-21 | 1.586008 |
| hsa-miR-2355-3p | 1.42E-20 | 2.43E-21 | -1.28648 |
| hsa-miR-432-5p | 1.30E-20 | 2.17E-21 | -1.84787 |
| hsa-miR-92a-3p | 1.12E-20 | 1.82E-21 | 0.735765 |
| hsa-miR-1224-5p | 3.10E-20 | 5.45E-21 | 1.747204 |
| hsa-miR-27a-3p | 3.01E-20 | 5.22E-21 | -0.65829 |
| hsa-miR-889-3p | 5.40E-20 | 9.62E-21 | -1.72673 |
| hsa-miR-181c-3p | 5.66E-20 | 1.02E-20 | -1.35658 |
| hsa-miR-431-5p | 8.14E-20 | 1.48E-20 | -1.82699 |
| hsa-miR-671-3p | 8.72E-20 | 1.64E-20 | -1.36497 |
| hsa-miR-100-5p | 8.72E-20 | 1.62E-20 | 1.565418 |
| hsa-miR-96-5p | 8.72E-20 | 1.63E-20 | 1.504981 |
| hsa-miR-483-3p | 9.54E-20 | 1.82E-20 | 2.290889 |
| hsa-miR-224-5p | 1.19E-19 | 2.29E-20 | -1.52804 |
| hsa-miR-382-5p | 1.50E-19 | 2.92E-20 | -1.66344 |
| hsa-miR-376a-3p | 3.94E-19 | 7.77E-20 | -1.75129 |
| hsa-miR-144-3p | 7.40E-19 | 1.47E-19 | 1.377081 |
| hsa-miR-28-5p | 1.05E-18 | 2.13E-19 | -1.16121 |
| hsa-miR-23a-3p | 7.91E-19 | 1.59E-19 | -0.61048 |
| hsa-miR-769-5p | 1.86E-18 | 3.81E-19 | -0.95139 |
| hsa-miR-370-3p | 2.07E-18 | 4.34E-19 | -1.80194 |
| hsa-miR-379-5p | 2.16E-18 | 4.57E-19 | -1.59931 |
| hsa-miR-664a-5p | 2.06E-18 | 4.29E-19 | -0.77533 |
| hsa-miR-369-5p | 2.49E-18 | 5.33E-19 | -1.7137 |
| hsa-miR-101-3p | 2.77E-18 | 5.98E-19 | 0.748329 |
| hsa-miR-628-5p | 4.45E-18 | 9.72E-19 | -1.18128 |
| hsa-miR-1277-3p | 4.84E-18 | 1.08E-18 | -1.31494 |
| hsa-miR-3200-3p | 6.12E-18 | 1.39E-18 | 1.576746 |
| hsa-miR-27b-3p | 4.81E-18 | 1.06E-18 | -0.67254 |
| hsa-miR-30a-5p | 5.73E-18 | 1.29E-18 | 0.85234 |
| hsa-miR-501-3p | 7.40E-18 | 1.69E-18 | 0.978094 |
| hsa-miR-26b-3p | 1.04E-17 | 2.45E-18 | -0.84217 |
| hsa-miR-423-3p | 8.98E-18 | 2.09E-18 | -0.92845 |
| hsa-miR-3184-5p | 8.98E-18 | 2.09E-18 | -0.92845 |
| hsa-miR-550a-3p | 1.16E-17 | 2.83E-18 | 1.694119 |
| hsa-miR-550b-2-5p | 1.16E-17 | 2.83E-18 | 1.694119 |
| hsa-miR-4433-5p | 1.16E-17 | 2.78E-18 | -1.54377 |
| hsa-miR-4433b-3p | 1.16E-17 | 2.78E-18 | -1.54369 |
| hsa-miR-26b-5p | 1.39E-17 | 3.42E-18 | -0.58827 |
| hsa-miR-205-5p | 2.22E-17 | 5.60E-18 | 1.665754 |
| hsa-miR-3591-3p | 1.89E-17 | 4.73E-18 | 1.803858 |
| hsa-miR-122-5p | 1.89E-17 | 4.73E-18 | 1.803857 |
| hsa-miR-378a-3p | 3.01E-17 | 7.66E-18 | 0.912226 |
| hsa-miR-215-5p | 3.78E-17 | 9.68E-18 | 1.491124 |
| hsa-miR-33a-5p | 7.31E-17 | 1.89E-17 | -0.98693 |
| hsa-miR-548a-3p | 8.22E-17 | 2.14E-17 | -1.21392 |
| hsa-let-7b-3p | 1.03E-16 | 2.71E-17 | 0.913406 |
| hsa-miR-203a | 1.03E-16 | 2.74E-17 | 1.595144 |
| hsa-miR-203b-5p | 1.03E-16 | 2.74E-17 | 1.595144 |
| hsa-miR-193b-5p | 1.09E-16 | 2.94E-17 | 1.757678 |
| hsa-miR-652-3p | 1.19E-16 | 3.22E-17 | -0.98618 |
| hsa-miR-10b-3p | 1.34E-16 | 3.65E-17 | 1.745529 |
| hsa-miR-361-3p | 1.94E-16 | 5.37E-17 | -0.63419 |
| hsa-miR-493-5p | 2.61E-16 | 7.29E-17 | -1.61245 |
| hsa-miR-369-3p | 3.15E-16 | 8.88E-17 | -1.64611 |
| hsa-miR-376c-3p | 3.33E-16 | 9.46E-17 | -1.69385 |
| hsa-miR-18b-3p | 4.68E-16 | 1.34E-16 | 1.537066 |
| hsa-miR-494-3p | 4.88E-16 | 1.41E-16 | -1.4715 |
| hsa-miR-374b-5p | 5.29E-16 | 1.55E-16 | -1.06704 |
| hsa-miR-374c-3p | 5.29E-16 | 1.55E-16 | -1.06704 |
| hsa-miR-556-3p | 6.41E-16 | 1.89E-16 | -1.20423 |
| hsa-miR-101-5p | 7.75E-16 | 2.30E-16 | 1.295187 |
| hsa-miR-6805-5p | 7.88E-16 | 2.35E-16 | 1.517676 |
| hsa-miR-885-5p | 8.58E-16 | 2.58E-16 | 2.042068 |
| hsa-miR-92b-3p | 9.32E-16 | 2.83E-16 | 0.920137 |
| hsa-miR-339-3p | 1.44E-15 | 4.39E-16 | -0.77588 |
| hsa-miR-493-3p | 1.66E-15 | 5.11E-16 | -1.48834 |
| hsa-miR-3605-3p | 2.95E-15 | 9.14E-16 | 1.135805 |
| hsa-miR-99a-5p | 5.60E-15 | 1.76E-15 | 0.973153 |
| hsa-miR-125b-5p | 4.84E-15 | 1.51E-15 | 0.931711 |
| hsa-miR-34a-5p | 7.23E-15 | 2.30E-15 | 1.036786 |
| hsa-miR-141-3p | 7.67E-15 | 2.45E-15 | 1.098719 |
| hsa-miR-3158-5p | 1.05E-14 | 3.37E-15 | 1.511917 |
| hsa-miR-95-3p | 1.07E-14 | 3.47E-15 | 1.03135 |
| hsa-miR-487b-3p | 1.17E-14 | 3.80E-15 | -1.54322 |
| hsa-miR-3158-3p | 1.38E-14 | 4.58E-15 | 1.492368 |
| hsa-miR-877-5p | 1.35E-14 | 4.45E-15 | -0.9404 |
| hsa-miR-636 | 1.54E-14 | 5.16E-15 | 1.537928 |
| hsa-miR-576-5p | 1.24E-14 | 4.07E-15 | 0.632885 |
| hsa-miR-1908-5p | 1.55E-14 | 5.23E-15 | -1.10893 |
| hsa-miR-642a-3p | 1.80E-14 | 6.14E-15 | 1.426922 |
| hsa-miR-642b-5p | 1.80E-14 | 6.14E-15 | 1.426922 |
| hsa-miR-6780a-5p | 1.92E-14 | 6.60E-15 | 1.172413 |
| hsa-miR-301a-3p | 2.67E-14 | 9.22E-15 | -0.85055 |
| hsa-miR-590-3p | 3.51E-14 | 1.22E-14 | -0.76473 |
| hsa-miR-323a-3p | 4.05E-14 | 1.41E-14 | -1.54746 |
| hsa-miR-491-5p | 5.15E-14 | 1.84E-14 | -1.0992 |
| hsa-miR-4433-3p | 4.10E-14 | 1.45E-14 | -1.4422 |
| hsa-miR-4433b-5p | 4.10E-14 | 1.45E-14 | -1.4422 |
| hsa-miR-937-3p | 5.95E-14 | 2.16E-14 | 1.27495 |
| hsa-miR-500a-3p | 5.54E-14 | 1.99E-14 | 0.798645 |
| hsa-miR-328-3p | 4.63E-14 | 1.65E-14 | -1.04955 |
| hsa-miR-3065-5p | 6.04E-14 | 2.20E-14 | -0.72295 |
| hsa-miR-338-3p | 6.09E-14 | 2.23E-14 | -0.72271 |
| hsa-miR-3138 | 7.65E-14 | 2.82E-14 | -0.93887 |
| hsa-miR-23b-3p | 1.13E-13 | 4.20E-14 | 0.613289 |
| hsa-miR-425-3p | 1.32E-13 | 4.94E-14 | -0.71554 |
| hsa-miR-3940-3p | 1.71E-13 | 6.44E-14 | 1.325972 |
| hsa-miR-760 | 1.80E-13 | 6.81E-14 | -1.007 |
| hsa-miR-365b-3p | 2.69E-13 | 1.03E-13 | 1.264101 |
| hsa-miR-365a-3p | 2.76E-13 | 1.06E-13 | 1.263289 |
| hsa-miR-218-5p | 2.91E-13 | 1.13E-13 | 1.502382 |
| hsa-miR-382-3p | 4.35E-13 | 1.70E-13 | -1.38148 |
| hsa-miR-323b-3p | 8.06E-13 | 3.18E-13 | -1.53666 |
| hsa-miR-484 | 7.24E-13 | 2.84E-13 | 0.807237 |
| hsa-miR-539-3p | 1.09E-12 | 4.33E-13 | -1.31849 |
| hsa-miR-579-3p | 1.20E-12 | 4.78E-13 | 1.163597 |
| hsa-miR-1307-5p | 1.50E-12 | 6.02E-13 | -0.79767 |
| hsa-miR-106b-5p | 1.50E-12 | 6.00E-13 | 0.695518 |
| hsa-miR-18b-5p | 3.96E-12 | 1.61E-12 | 0.976057 |
| hsa-miR-337-5p | 4.08E-12 | 1.67E-12 | -1.44326 |
| hsa-miR-5001-3p | 5.92E-12 | 2.44E-12 | 1.053293 |
| hsa-miR-136-3p | 6.22E-12 | 2.58E-12 | -1.27404 |
| hsa-miR-532-5p | 5.07E-12 | 2.08E-12 | 0.599297 |
| hsa-miR-485-5p | 7.39E-12 | 3.09E-12 | -1.33791 |
| hsa-miR-1185-1-3p | 8.83E-12 | 3.71E-12 | -1.27708 |
| hsa-miR-106a-3p | 9.59E-12 | 4.07E-12 | 1.053412 |
| hsa-miR-206 | 9.01E-12 | 3.80E-12 | 1.610648 |
| hsa-miR-885-3p | 1.28E-11 | 5.44E-12 | 1.639069 |
| hsa-miR-139-3p | 1.73E-11 | 7.41E-12 | -0.85743 |
| hsa-miR-329-3p | 2.23E-11 | 9.58E-12 | -1.35259 |
| hsa-miR-3143 | 2.36E-11 | 1.02E-11 | 0.911353 |
| hsa-miR-98-3p | 3.23E-11 | 1.40E-11 | -0.83473 |
| hsa-miR-3688-3p | 4.49E-11 | 2.00E-11 | 0.992736 |
| hsa-miR-3688-5p | 4.49E-11 | 2.00E-11 | 0.992736 |
| hsa-miR-574-3p | 3.75E-11 | 1.64E-11 | -0.65858 |
| hsa-miR-25-5p | 4.59E-11 | 2.05E-11 | 0.79679 |
| hsa-miR-29b-3p | 3.75E-11 | 1.64E-11 | 0.687615 |
| hsa-miR-20b-5p | 4.47E-11 | 1.97E-11 | 0.749258 |
| hsa-miR-4532 | 6.43E-11 | 2.89E-11 | 1.398562 |
| hsa-miR-153-3p | 7.15E-11 | 3.23E-11 | 1.131018 |
| hsa-miR-320e | 1.14E-10 | 5.21E-11 | 1.121132 |
| hsa-miR-214-5p | 1.17E-10 | 5.38E-11 | -0.88748 |
| hsa-miR-3120-3p | 1.17E-10 | 5.38E-11 | -0.88748 |
| hsa-miR-150-5p | 8.31E-11 | 3.77E-11 | 0.824025 |
| hsa-miR-133a-3p | 1.28E-10 | 5.93E-11 | -1.11171 |
| hsa-miR-21-3p | 1.37E-10 | 6.35E-11 | -0.72233 |
| hsa-miR-6842-3p | 1.61E-10 | 7.56E-11 | -0.84804 |
| hsa-miR-651-5p | 1.53E-10 | 7.14E-11 | 0.651881 |
| hsa-miR-4685-3p | 2.05E-10 | 9.65E-11 | 1.198465 |
| hsa-miR-582-3p | 2.12E-10 | 1.00E-10 | 1.327642 |
| hsa-miR-22-5p | 2.21E-10 | 1.05E-10 | -0.73787 |
| hsa-miR-548j-5p | 3.70E-10 | 1.76E-10 | -0.91289 |
| hsa-miR-2116-3p | 4.90E-10 | 2.36E-10 | 1.096006 |
| hsa-miR-374a-5p | 3.90E-10 | 1.87E-10 | -0.65589 |
| hsa-miR-154-5p | 5.44E-10 | 2.63E-10 | -1.15646 |
| hsa-miR-3140-3p | 6.75E-10 | 3.28E-10 | 0.946655 |
| hsa-let-7i-3p | 7.18E-10 | 3.50E-10 | 0.780506 |
| hsa-miR-6852-3p | 1.25E-09 | 6.09E-10 | -0.93235 |
| hsa-miR-411-5p | 1.96E-09 | 9.62E-10 | -1.13729 |
| hsa-miR-624-5p | 3.13E-09 | 1.55E-09 | 0.906673 |
| hsa-miR-377-3p | 3.77E-09 | 1.87E-09 | -1.06602 |
| hsa-miR-150-3p | 3.99E-09 | 1.99E-09 | 1.046744 |
| hsa-miR-5187-5p | 4.38E-09 | 2.19E-09 | -0.83619 |
| hsa-miR-30d-3p | 4.67E-09 | 2.36E-09 | -0.6822 |
| hsa-miR-4772-3p | 5.48E-09 | 2.79E-09 | 1.150784 |
| hsa-miR-1271-5p | 5.54E-09 | 2.83E-09 | -0.83888 |
| hsa-miR-485-3p | 5.08E-09 | 2.57E-09 | -1.45085 |
| hsa-miR-337-3p | 7.91E-09 | 4.06E-09 | -1.04983 |
| hsa-miR-3611 | 8.52E-09 | 4.39E-09 | 0.83988 |
| hsa-miR-10a-3p | 9.42E-09 | 4.87E-09 | 0.856636 |
| hsa-miR-200a-3p | 1.02E-08 | 5.31E-09 | 0.873204 |
| hsa-miR-210-3p | 1.19E-08 | 6.19E-09 | 0.786154 |
| hsa-miR-3679-5p | 1.20E-08 | 6.28E-09 | -0.77879 |
| hsa-miR-873-5p | 2.55E-08 | 1.35E-08 | 1.124472 |
| hsa-miR-331-3p | 2.56E-08 | 1.35E-08 | -0.74372 |
| hsa-miR-570-3p | 3.47E-08 | 1.84E-08 | 0.864768 |
| hsa-miR-320d | 3.69E-08 | 1.97E-08 | 0.757995 |
| hsa-miR-151a-5p | 3.90E-08 | 2.09E-08 | -0.8034 |
| hsa-miR-548l | 4.58E-08 | 2.48E-08 | 0.759828 |
| hsa-miR-4429 | 4.47E-08 | 2.40E-08 | 0.810368 |
| hsa-miR-4662a-5p | 5.40E-08 | 2.95E-08 | -0.84506 |
| hsa-miR-4662b | 5.40E-08 | 2.95E-08 | -0.84506 |
| hsa-let-7g-3p | 5.74E-08 | 3.16E-08 | 0.844104 |
| hsa-miR-190a-5p | 5.40E-08 | 2.95E-08 | 0.790866 |
| hsa-miR-92b-5p | 1.06E-07 | 5.94E-08 | 0.726112 |
| hsa-miR-503-5p | 9.78E-08 | 5.43E-08 | 0.586258 |
| hsa-miR-548o-3p | 1.44E-07 | 8.15E-08 | 0.80378 |
| hsa-miR-424-5p | 1.17E-07 | 6.57E-08 | 0.599204 |
| hsa-miR-221-5p | 1.59E-07 | 9.06E-08 | -0.77844 |
| hsa-miR-6735-5p | 1.68E-07 | 9.58E-08 | 0.730728 |
| hsa-miR-211-5p | 1.75E-07 | 9.99E-08 | 0.994568 |
| hsa-miR-29b-2-5p | 1.77E-07 | 1.02E-07 | 0.812748 |
| hsa-miR-7976 | 2.26E-07 | 1.30E-07 | 0.742292 |
| hsa-miR-766-3p | 2.32E-07 | 1.35E-07 | -0.83868 |
| hsa-miR-499a-5p | 3.16E-07 | 1.85E-07 | 0.81538 |
| hsa-miR-499b-3p | 3.16E-07 | 1.85E-07 | 0.81538 |
| hsa-miR-3157-5p | 3.29E-07 | 1.93E-07 | 0.710508 |
| hsa-miR-18a-3p | 4.39E-07 | 2.58E-07 | 0.783722 |
| hsa-miR-3605-5p | 4.82E-07 | 2.85E-07 | 0.716416 |
| hsa-miR-589-3p | 8.71E-07 | 5.19E-07 | 0.68928 |
| hsa-miR-136-5p | 8.91E-07 | 5.32E-07 | -0.91183 |
| hsa-miR-181d-5p | 1.03E-06 | 6.21E-07 | -0.69728 |
| hsa-miR-1270 | 1.06E-06 | 6.38E-07 | 0.647825 |
| hsa-miR-664b-5p | 1.18E-06 | 7.17E-07 | -0.66328 |
| hsa-miR-3913-3p | 1.89E-06 | 1.16E-06 | 0.642953 |
| hsa-miR-3913-5p | 1.89E-06 | 1.16E-06 | 0.642953 |
| hsa-miR-376b-3p | 2.10E-06 | 1.29E-06 | -0.85111 |
| hsa-miR-495-3p | 2.33E-06 | 1.44E-06 | -0.83508 |
| hsa-miR-766-5p | 3.15E-06 | 1.95E-06 | -0.65371 |
| hsa-miR-4742-3p | 4.38E-06 | 2.73E-06 | 0.715218 |
| hsa-miR-548k | 5.44E-06 | 3.40E-06 | 0.666181 |
| hsa-miR-7706 | 7.40E-06 | 4.68E-06 | 0.616806 |
| hsa-miR-3173-5p | 7.25E-06 | 4.56E-06 | 0.678395 |
| hsa-miR-3127-5p | 7.97E-06 | 5.10E-06 | 0.618076 |
| hsa-miR-99b-3p | 8.05E-06 | 5.18E-06 | -0.70036 |
| hsa-miR-181c-5p | 1.16E-05 | 7.52E-06 | -0.70135 |
| hsa-miR-196a-5p | 1.62E-05 | 1.06E-05 | 0.893622 |
| hsa-miR-4659a-3p | 1.71E-05 | 1.13E-05 | 0.61306 |
| hsa-miR-4659b-5p | 1.71E-05 | 1.13E-05 | 0.61306 |
| hsa-miR-543 | 0.000147 | 0.0001 | -0.67989 |
| hsa-miR-6511b-3p | 0.000153 | 0.000105 | 0.636656 |
| hsa-miR-378a-5p | 0.000171 | 0.000118 | 0.610759 |
| hsa-miR-6803-3p | 0.000294 | 0.000208 | 0.650744 |
